# Musculoskeletal actuators with programmable morphology and tunable dynamics

**DOI:** 10.1126/sciadv.aeg4524

**Published:** 2026-07-29

**Authors:** Shiwei Xu, Chuanqi Zang, Zhenjia Tang, Ruoxi Yang, Liya Liu, Jinzhe Peng, Wenbo Pang, Zehao Gao, Xiaonan Hu, Wei Shen, Zhi Liu, Mingjing Qi, Renheng Bo, Yihui Zhang

**Affiliations:** ^1^ Applied Mechanics Laboratory, Department of Engineering Mechanics, Tsinghua University, Beijing 100084, P.R. China.; ^2^ State Key Laboratory of Flexible Electronics Technology, Tsinghua University, Beijing 100084, P.R. China.; ^3^ School of Energy and Power Engineering, Beihang University, Beijing 100191, P.R. China.; ^4^ Mechano-X institute, Tsinghua University, Beijing 100084, P.R. China.

## Abstract

Inspired by natural species exploiting morphological changes to adapt to complex environments, morphable robots have emerged as a frontier of robots with embodied intelligence, particularly for multimodal and cross-domain locomotion. However, the development of small-scale flexible actuators with dual programmability in morphology and dynamics remains elusive, hindering miniaturization of untethered morphable robots. Inspired by biological musculoskeletal systems, we present artificial musculoskeletal actuators constructed with serially connected morphable skeleton modules and stiffness-tunable muscle modules, enabling programming of both morphology and dynamics. The muscle module exploits a multilayer PDMS-based dielectric elastomer and an electrothermally actuated shape memory polymer, allowing modulation of resonant frequency, resonant vibration amplitude, and actuation force. Geometrically sophisticated musculoskeletal actuators are constructed to reproduce complex dynamic behaviors of species like woodpecker and scorpion. Demonstrations of an untethered sugar glider–inspired multimodal small-scale robot and a morphable small-scale robot with quadruped and humanoid locomotion modes suggest promising applications of proposed actuators.

## Teaser

Musculoskeletal actuators with programmable morphology and dynamics for morphable small-scale robots.

## INTRODUCTION

Many natural species possess the multimodal locomotion capability by actively adjusting their body morphologies, which offers enhanced adaptability to complex living environments. Inspired by this principle, morphable robots have emerged as a promising class of multimodal robots capable of traversing diverse terrains and operating across diverse physical domains (*1*, *2*). Conceptually, these morphable robots can be decomposed into two functional modules, namely, the transformation module that defines the overall robot configuration and the locomotion module that enables the robot movement under that configuration, although these two functions do not necessarily need to be realized as physically separate components. Notably, an important application scenario of the morphable robots is the locomotion and exploration in natural or open environments, which represents the key focus of the current study. Magnetically actuated robots (*3*–*5*), which offer remarkable morphing and locomotion capabilities under externally applied magnetic fields in confined biomedical settings, are therefore not considered in this context. In morphable robots, the transformation module is commonly realized using servo motors (*6*–*9*) or flexible actuators (*10*–*12*) capable of shape morphing and locking, whereas the locomotion module typically uses direct current (dc) or servo motors (*6*–*12*) to achieve rapid or continuous movement, therefore resulting in increased system complexity and challenges in miniaturization. This has motivated the exploration of soft active materials (*13*–*17*) and flexible structures (*18*–*30*) to enable the transformation and locomotion of morphable robots.

Consequently, a variety of high-performance actuators have been developed for morphable robots (*31*–*34*). Examples include fast-response actuators [such as dielectric elastomer actuators (DEAs) (*35*–*39*) and hydraulically amplified self-healing electrostatic (HASEL) actuators (*13*, *40*, *41*)] and flexible actuators capable of evident morphological transformations [such as those based on liquid crystal elastomers (LCEs) (*42*–*46*)]. While these actuators offer fast-response speeds, large deformations, and lightweight designs, they still face challenges when used to construct morphable small-scale robots (e.g., lateral size <10 cm). First, for the robotic system with a single type of actuator, the morphological transformation and fast locomotion are often intrinsically coupled and difficult to realize simultaneously. Second, for agile locomotion, DEAs and HASEL actuators typically exhibit fixed or weakly tunable dynamic characteristics (e.g., resonant frequency). Their requirement for kilovolt-level alternating-current driving voltages further poses a challenge for untethered operation, and many existing systems rely on external power supplies (*47*–*49*) or material-level strategies (*38*, *50*, *51*) to reduce operating voltages, which may compromise actuation performances. Because of these challenges, the development of small-scale actuators (e.g., lateral size <5 cm) with dual programmability in morphology and dynamics for untethered small-scale robots remains elusive.

In natural musculoskeletal systems, morphological transformation and dynamic actuation arise from the coordinated interaction between skeletal structures and muscles. In general, large-scale changes in body configuration follow from muscle-driven reconfiguration of skeletal structures and joints, whereas dynamic actuation mainly relies on forces and deformations generated by muscles, whose effective stiffness and response speed are regulated through neural signals. Even in small mammals (e.g., sugar gliders and flying squirrels) and insects (e.g., locusts), both the morphological transformation and the fast-response locomotion can be achieved through the coordination of their musculoskeletal systems. Inspired by this functional principle, we propose a design concept of artificial musculoskeletal actuators constructed with serially connected morphable skeleton modules and stiffness-tunable muscle modules, which enable independent programming of morphology and dynamic behavior. In particular, the muscle module integrates a multilayer polydimethylsiloxane (PDMS)–based dielectric elastomer (DE) with an electrothermally actuated shape memory polymer (SMP) capable of stiffness modulation. Its dynamic characteristics—including resonant frequency, resonant vibration amplitude, and actuation force—can be actively modulated through the stiffness variation. The skeleton module adopts a synergistic material-structure design of SMP and LCE reported in our previous work (*11*), allowing the shape morphing and locking. Both the muscle and the skeleton modules are electrically controlled, thereby simplifying the control complexity. Building on this design concept, geometrically sophisticated musculoskeletal actuators are constructed, enabling the reproduction of complex dynamic behaviors of natural species (e.g., human, peacock, woodpecker, and scorpion). Furthermore, an untethered sugar glider-inspired morphable small-scale robot (5 cm by 2 cm by 1.5 cm in the undeployed state; 6.4 g) based on proposed musculoskeletal actuators and a lightweight control module (generating low-to-mid-frequency voltages up to ∼9.5 kV) is demonstrated, exhibiting fast terrestrial locomotion [e.g., 1.1 body length (BL)/s], cargo carrying (e.g., 3.6 g), liquid-surface swimming (e.g., 0.37 BL/s), and aerial gliding. Notably, the actuation system of this robot, capable of complex morphological changes and tunable dynamics, is composed entirely of soft active materials, which has been challenging to realize in untethered morphable small-scale robots. This study establishes a body-centric approach to morphable small-scale robots, in which the coordination of morphological change and dynamic response plays a central role in enabling multimodal locomotion/operation capabilities.

## RESULTS

### Design concept and demonstration of musculoskeletal actuators

**Fig. 1.**
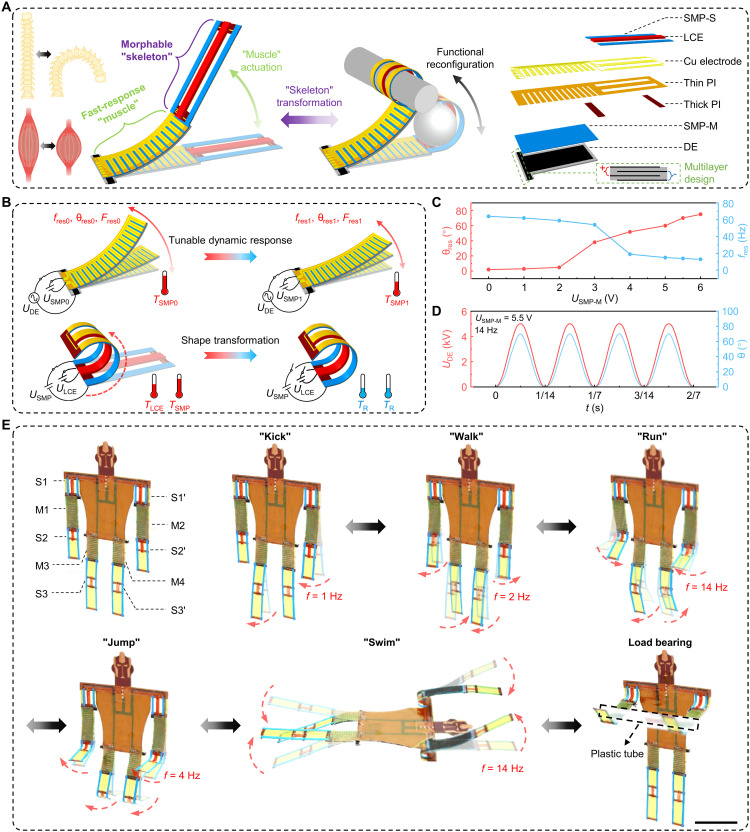
Design concept, operating principle, and demonstrations of musculoskeletal actuators. (**A**) Illustration and exploded view of the basic musculoskeletal actuator that offers dual programmability in morphology and dynamics. (**B**) Schematic illustration of the muscle module with tunable dynamic characteristics and the skeleton module capable of shape morphing and subsequent locking. The tunability of dynamic characteristics mainly results from the variable stiffness of SMP, while the capability of shape morphing and locking originates from the synergistic effect of LCE and SMP. (**C**) Influence of *U*_SMP-M_ (the voltage applied to the SMP layer within the muscle module) on the resonant frequency (*f*_res_) and the corresponding resonant bending angle (θ_res_) of the muscle module. (**D**) Time-resolved bending response of the muscle module driven by a 14-Hz sinusoidal voltage applied to the multilayer PDMS-based DE, with a peak of *U*_DE_ = 5 kV and a dc voltage of *U*_SMP-M_ = 5.5 V. (**E**) Optical images of a centimeter-scale human-shaped musculoskeletal actuator constructed using four muscle modules (M1 to M4) and six skeleton modules (S1 to S3 and S1′ to S3′) connected in series, demonstrating human-like actions (including kick, walk, run, jump, and swim) and load-bearing capability. The prime symbol (′) indicates the symmetric counterpart of skeleton module. Scale bar, 2 cm. Photo credit: S.X., Tsinghua University. PI, polyimide.

**Fig. 2.**
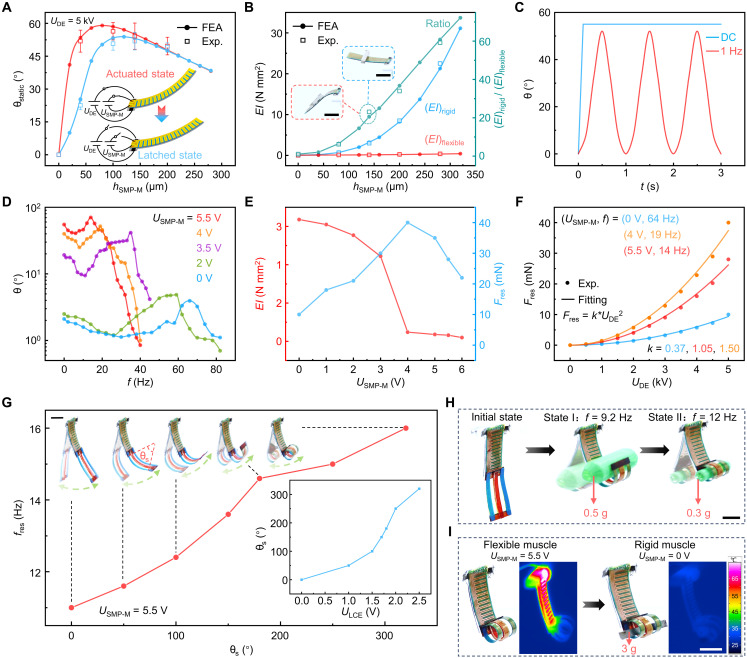
Performances of the basic musculoskeletal actuator. (**A**) Static bending angles of the muscle module in the actuated state (red) and the latched state (blue) as a function of the SMP layer thickness (*h*_SMP-M_), according to the FEA and experiments. Here, a dc HV is applied to the DE layer. Data are presented as mean values, and error bars are s.d. values from three independent samples. (**B**) Influence of *h*_SMP-M_ on the bending stiffness of the muscle module in the rigid state [*U*_SMP-M_ = 0 V; (*EI*)_rigid_] and flexible state [*U*_SMP-M_ = 5.5 V; (*EI*)_flexible_]. Scale bars, 5 mm. (**C**) Comparison of the time-dependent muscle deformation driven by a 14-Hz sinusoidal voltage and by a dc voltage, with *U*_SMP-M_ = 5.5 V. (**D**) Dependence of the bending angle amplitude on the actuation frequency when different *U*_SMP-M_ values are adopted. (**E**) Bending stiffness and resonant actuation force of the muscle module versus the voltage *U*_SMP-M_. (**F**) Resonant actuation force of the muscle module as a function of the voltage applied to the DE layer under different muscle stiffness states, indicating an approximately quadratic relationship. (**G**) Influence of skeleton configuration (with different bending angles) on the resonant frequency of the basic musculoskeletal actuator (with *U*_SMP-M_ = 5.5 V). The inset shows the relationship between the bending angle of the skeleton module and the voltage applied to the LCE layer. Scale bar, 5 mm. (**H**) Demonstration of the shape transformation to carry differently sized objects. Scale bar, 5 mm. (**I**) Demonstration of static load-bearing capability, achieved by dc-driven deformation of the flexible muscle module, followed by cooling-induced shape locking and subsequent load bearing. Scale bar, 5 mm. Photo credit: S.X., Tsinghua University.

**Fig. 3.**
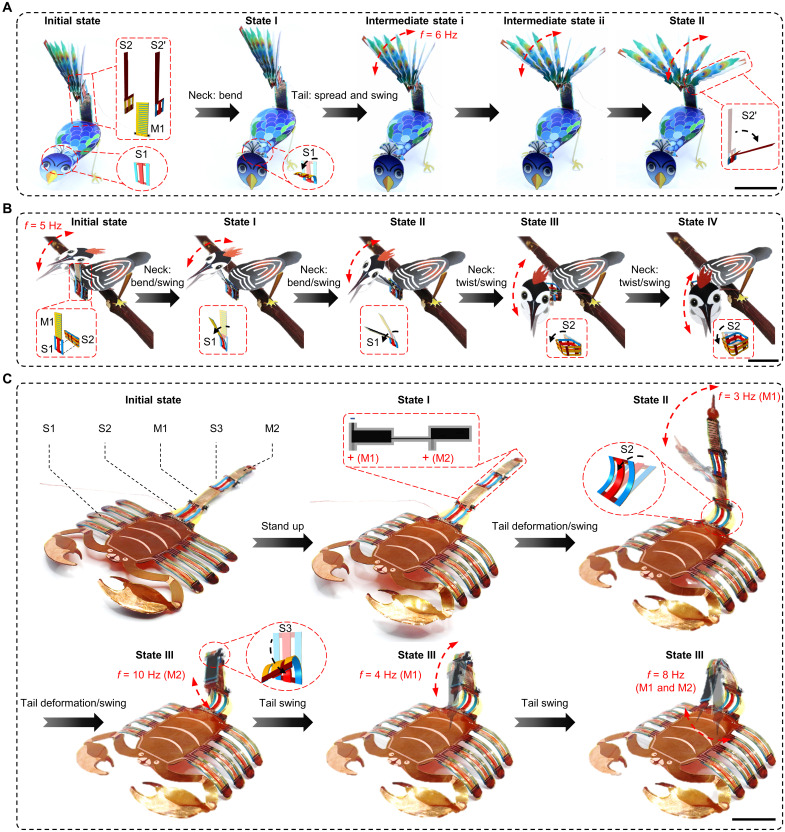
Biomimetic musculoskeletal actuators exhibiting versatile morphological changes and diverse dynamic responses. (**A**) A peacock-like actuator demonstrating neck bending, followed by tail spreading, along with concurrent back-and-forth swinging. Scale bar, 2 cm. (**B**) Woodpecker-like actuator with agile neck motions, enabling versatile woodpecking behaviors. Scale bar, 2 cm. (**C**) Scorpion-like actuator demonstrating complex tail-swinging motions through synergistic control of multiple muscle and skeleton modules. Scale bar, 2 cm. Photo credit: S.X., Tsinghua University.

**Fig. 4.**
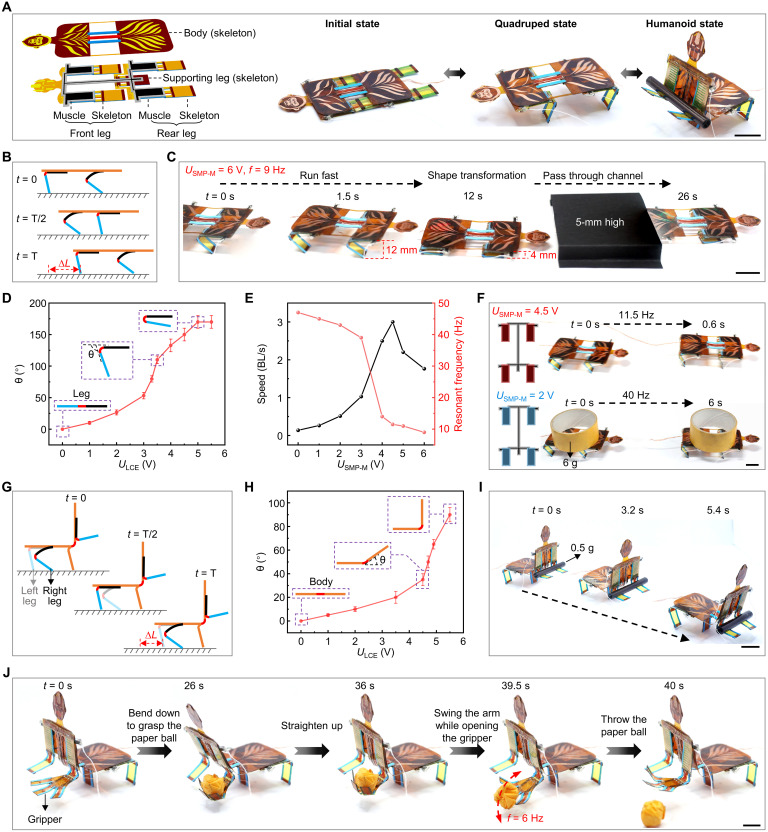
Morphable small-scale robot capable of transforming between quadruped and humanoid states. (**A**) Design and illustration of the morphable robot. The left panel shows the exploded view of the robot. The right panel displays the transformation from the initial planar state to the quadruped state and then the humanoid state. Scale bar, 1 cm. (**B**) Schematic illustration of the quadruped locomotion achieved by alternating actuation of front and rear muscle modules. (**C**) Optical images of the robot passing through a low-height channel (5 mm) enabled by lowering the height via leg deformation. Scale bar, 1 cm. (**D**) Dependence of the leg bending angle on the voltage applied to the LCE layer within the skeleton module. Data are presented as mean values, and error bars are SD values from three independent samples. (**E**) Influence of muscle stiffness (tuned by the *U*_SMP-M_) on the maximum quadruped speed and the resonant frequency. (**F**) Comparison of the locomotion performance under two different muscle stiffness states, where lower stiffness (*U*_SMP-M_ = 4.5 V) enables faster movement, and higher stiffness (*U*_SMP-M_ = 2 V) allows larger load-carrying capacity. Scale bar, 1 cm. (**G**) Schematic illustration of the humanoid locomotion achieved by alternating actuation of the two rear muscle modules. (**H**) Dependence of the bending angle of the top skeleton module on the voltage applied to its LCE layer. Data are presented as mean values, and error bars are s.d. values from three independent samples. (**I**) Optical images of the robot walking with the humanoid locomotion, while carrying a black plastic tube using the two arms. Scale bar, 1 cm. (**J**) Demonstration of the functional extension by incorporating an additional gripper skeleton module. Scale bar, 1 cm. Photo credit: S.X., Tsinghua University.

The design concept, working principle, and construction of a basic musculoskeletal actuator with dual programmability in morphology and dynamics are illustrated in Fig. 1, A and B, figs. S1 to S3, and movies S1 to S3. The actuator consists of a muscle module with tunable dynamic characteristics and a serially connected skeleton module capable of shape morphing and locking (Fig. 1A). Inspired by fiber-shaped layout of biological muscle, the muscle module comprises a serpentine heating electrode layer (2-μm Cu), a thin polyimide (PI) support layer (15 μm) with kirigami pattern to minimize the bending stiffness, an SMP layer (SMP-M, 150 μm), and a DE layer composed of multilayer unstretched PDMS (100 μm per layer) and flexible electrode (carbon grease). In the muscle module, the DE layer serves for the dynamic actuation, and the SMP layer serves to regulate the overall muscle stiffness (Fig. 1B). The actuation response of the DE can be controlled by the amplitude and frequency of the applied high voltage (HV) *U*_DE_, which is on the kilovolt level in this study. Notably, the peak value of the applied HV is 5 kV for most cases in Figs. 1 to 4 unless otherwise specified. The multilayer PDMS architecture combined with ultrathin flexible electrodes enables enhanced actuation force without increasing the driving voltage (fig. S2). The elastic modulus of the SMP layer can be reversibly modulated through the electrothermal heating induced by the voltage (*U*_SMP-M_) applied to the heating electrode (figs. S4 and S5), exhibiting an evident change from approximately 1824 MPa at 25°C to 2.42 MPa at 60°C. According to the classical vibration theory, variation in bending stiffness directly alters the dynamic response of a cantilever beam. Consequently, key dynamic characteristics of the muscle module—including resonant frequency (*f*_res_), resonant bending angle (θ_res_), and actuation force (*F*_res_)—can be actively tuned by *U*_SMP-M_. Specifically, the increase of *U*_SMP-M_ leads to a decrease in *f*_res_ and an increase in θ_res_, as shown in Fig. 1C. To avoid a noticeable thermal expansion of the PDMS layer, *U*_SMP-M_ is maintained within an appropriate range (e.g., <5.5 V), and the actuation frequency range discussed in this study only covers the first-order resonant frequency. In addition, the muscle module exhibits a fast response under 14-Hz sinusoidal excitation applied to the DE layer, which can be attributed to the relatively weak viscoelasticity of the unstretched PDMS (Fig. 1D).

The skeleton module adopts a heterogeneously integrated design of SMP and LCE reported in our previous work (*11*), with a simplified layer construction. It consists of an active layer [with separated LCE (400 μm) and SMP (SMP-S, 150 μm) ribbons], a serpentine heating electrode layer (2-μm Cu), a PI support layer (15 μm), and a thick PI layer (250 μm) ensuring coordinated deformation of LCE and SMP ribbons (Fig. 1A). Notably, SMP-S and SMP-M exhibit slightly different glass transition temperatures (*T*_g_ = 63° and 55°C, respectively) (fig. S4). The LCE ribbon functions as the active actuation part, and the SMP ribbons serve to lock the deformed configuration right after the actuation. Through the synergistic control of voltages applied to the heating electrodes of LCE and SMP ribbons, the skeleton module enables controlled shape morphing and locking at any intermediate deformation state (Fig. 1B and movie S3). The muscle and skeleton modules are serially connected, with the Cu electrode and thin PI layer fabricated in a single process, allowing the basic musculoskeletal actuator to provide both capabilities of shape transformation and dynamic actuation. Consequently, the basic musculoskeletal actuator supports dual programmability in morphology and dynamics in small-scale actuation systems (3 cm by 0.8 cm).

Through the rational design of serially connected muscle and skeleton modules, geometrically sophisticated musculoskeletal actuators with versatile morphological changes and diverse dynamic responses can be constructed. Here, a centimeter-scale human-shaped musculoskeletal actuator was developed, comprising four individually controlled muscle modules (“M1” to “M4”) and three pairs of independently controlled skeleton modules (“S1”/“S1,′” “S2”/“S2′,” and “S3”/“S3′”) (Fig. 1E and fig. S6). Several representative human actions and load-bearing capability are demonstrated, where kick and walk are performed in the initial planar state, and the other three actions (run, jump, and swim) are realized upon distinct morphologies enabled by skeleton reconfigurations (movie S4). By coordinating the actuation sequence of selected muscle modules (“M2” and “M3”), a kick-like action was produced via simultaneous activation of left-arm and right-leg muscles, while walking was achieved by alternating actuation of two diagonal muscle pairs (M1/M4 and M2/M3). To mimic the run action, the skeleton modules (S2, S2′, S3, and S3′) first underwent a controlled shape transformation to establish an appropriate body posture, followed by alternating actuation of the two diagonal muscle pairs. A similar shape transformation process was then performed, after which simultaneous actuation of all four muscle modules enabled the jump action. The swim action could be achieved in a similar manner. In addition to dynamic actions, the musculoskeletal actuator also offers static load-bearing capability, as demonstrated by carrying a plastic tube when both the muscle and skeleton modules were in rigid states.

### Designs and performances of the basic musculoskeletal actuators

For the basic musculoskeletal actuator, the design optimization mainly focuses on the muscle module, as illustrated in Fig. 2 (A and B) and figs. S7 and S8. Note that the key structural parameters of the skeleton module are fixed and adopt the optimal values obtained through combined theoretical and finite element analysis (FEA) studies (*11*), as given in the last section. Both the thicknesses of the SMP layer and the DE layer have an evident influence on the dynamic response of the muscle module. To carry out the parameter optimization, the thickness of the DE layer is fixed at an ∼300 μm (i.e., three stacked PDMS membranes). As shown in Fig. 2A and fig. S7, an optimal thickness *h*_SMP-M_ is required to achieve a large static bending angle. Here, the actuated state corresponds to the muscle operating under a dc HV of 5 kV together with *U*_SMP-M_ = 5.5 V, while the latched state refers to the deformed configuration in which the SMP layer is cooled below its *T*_g_ and the DE layer is no longer supplied with dc HV (fig. S9 and movie S2). Owing to the remarkable softening of the SMP above *T*_g_, the muscle module exhibits an evident stiffness variation between room temperature and elevated temperature (e.g., 25° and 60°C) (Fig. 2B). To maximize the tunability of dynamic response, a sufficiently large bending stiffness ratio between the rigid (*U*_SMP-M_ = 0 V) and the flexible state (*U*_SMP-M_ = 5.5 V) is essential. Balancing the considerations of bending stiffness ratio and deformation amplitude, the thickness of the SMP layer was selected as 0.15 mm in most of the subsequent experiments.

Figure 2C and fig. S10 present the comparison of the dynamic bending responses for the fabricated muscle module driven by a 14-Hz sinusoidal HV and a dc HV. The amplitude-frequency response curves of the muscle module at representative stiffness levels are provided in Fig. 2D. As *U*_SMP-M_ increases from 0 V to 5.5 V, the muscle stiffness decreases markedly, resulting in a decrease in the resonant frequency (from 64 to 14 Hz) and a substantial increase in the resonant bending angle (from 2.5° to 75°) (Fig. 2D and movie S2). Meanwhile, owing to the competing effects of stiffness reduction and deformation increase, the resonant actuation force exhibits a nonmonotonic dependence on *U*_SMP-M_, where a peak actuation force can be observed at *U*_SMP-M_ = 4 V (Fig. 2E and fig. S11). In addition, both the resonant bending angle and actuation force show an approximately quadratic dependence on *U*_DE_, as shown in Fig. 2F and fig. S7. The actuation force can be enlarged by synergistically increasing the thicknesses of SMP and DE layers, providing adaptability for versatile application requirements. The muscle module maintains stable actuation performance over repeated operation (1000 actuation cycles in fig. S12). In addition, potential failure modes of the actuator include dielectric breakdown in the multilayer PDMS, interface delamination within the multilayer PDMS or at the PDMS/SMP and SMP/heating layer interfaces, and crack initiation/propagation in the SMP layer under repeated thermomechanical cycling. By serially connecting the muscle module with the as-designed skeleton module, the basic musculoskeletal actuator was developed, enabling coordinated programming of morphology and dynamic behavior (figs. S13 to S15 and movie S3). In addition to the dominant influence of muscle stiffness on the resonant frequency, the overall configuration of the actuator, controlled by skeleton deformation, also plays a non-negligible role. As the bending angle of the skeleton module increases under voltage-controlled actuation of the LCE layer, the resonant frequency of the overall actuator rises from 11 to 16 Hz when the muscle module is in the flexible state (*U*_SMP-M_ = 5.5 V) (Fig. 2G). Figure 2H and movie S3 further demonstrate that the shape transformation enables the actuator to grasp objects of different geometries. Owing to the relatively high stiffness (at room temperature) of SMP layers within the skeleton and muscle modules, the actuator has a relatively high load-bearing capacity. For example, when both modules are in their latched deformation states, the actuator (0.18 g) can bear a load of 3 g (Fig. 2I).

Overall, the actuator does not maximize all performance metrics simultaneously but instead enables a balance among deformation amplitude, force output, load-bearing capability, and energy consumption. As shown in the above results, muscle stiffness strongly affects resonant frequency, amplitude, force output, and load-bearing behavior, whereas skeleton morphology mainly changes the geometry and contributes less strongly to dynamic tuning. Although the energy consumption generally increases with increasing actuation frequency, the largest deformation amplitude and output force are achieved only when the actuator is driven near resonance. Compared with conventional DEAs, HASEL, and LCE-based actuators, the present actuator combines the functions of morphology reconfiguration, stiffness modulation, and rapid actuation within one architecture, thereby enabling dual programmability in both morphology and dynamics. This multifunctionality comes at the cost of additional electrothermal energy consumption but provides tunable actuation force and enhanced load-carrying capability through stiffness adjustment.

### Actuators with versatile morphological changes and diverse dynamic responses

Through rational design of serially connected skeleton and muscle modules, complex musculoskeletal actuators with versatile morphological changes and diverse dynamic responses can be constructed, enabling the reproduction of complex dynamic behavior of natural species (Fig. 3 and movie S5). Peacocks can deploy their ornate tails to a very large extent while generating back-and-forth tail swinging to attract mates. Figure 3A presents a peacock-like actuator composed of three skeleton modules, a muscle module, and a lightweight cardboard substrate (200 μm) with printed cartoon pattern to mimic the peacock. In this actuator, the head skeleton module (S1) controls head orientation, the paired tail skeletons (S2 and S2′) enable tail deployment, and the muscle module facilitates rapid tail swinging. Starting from the initial state, the actuator first lowered its head and then spread and swung the tail simultaneously under 6-Hz muscle actuation. Woodpeckers rely on their specialized body structure, particularly the highly agile necks, to precisely control force and orientation during pecking. Figure 3B shows a woodpecker-like actuator consisting of two skeleton modules (S1 and S2), a muscle module (M1), and a patterned cardboard substrate. The skeleton modules S1 and S2 (fig. S16) controls head bending and rotation, respectively, while the muscle module M1 allows the head to peck. Through sequential morphing and locking of S1 and S2, the actuator transitioned from the initial state to a few representative configurations (states I, II, III, and IV). In this set of experiments, a 5-Hz muscle actuation was adopted to drive the pecking.

The tails of scorpions can allow large-amplitude and high-frequency motions that are essential for both defense and predation. Figure 3C and fig. S17 illustrate a scorpion-like actuator equipped with serially integrated muscle and skeleton modules to reproduce these complex tail behaviors. Here, the leg structure consists of eight skeleton modules (S1) with shared heating circuits such that their shape morphing and locking are controlled in a concurrent manner. The tail is formed by serially connected skeleton modules (S2 and S3) and muscle modules (M1 and M2) arranged in an alternating sequence. Coordinated morphing and locking of leg skeleton modules transformed the actuator from a planar state into a standing posture (state I). The subsequent bending of S2 elevated the tail into an upright position, corresponding to state II. At this morphology, driving M1 at 3 Hz excited a resonance of the entire actuator, resulting in a forward-backward tail swinging with an amplitude of ∼2.9 cm for the tail end. Then, coupled bending-twisting deformations of S3 transformed the actuator into state III, leading to an oblique bending configuration of the tail. At this morphology, different dynamic behaviors can be triggered by selectively activating the two muscle modules. Specifically, driving M2 at 10 Hz induced a localized resonant motion concentrated at the tail tip (with an amplitude of ∼0.4 cm for the tail end), whereas driving M1 at 4 Hz generated large-amplitude bending of the entire tail (amplitude ∼1.5 cm). When both muscle modules are simultaneously driven at 8 Hz, the tail exhibited a combined bending and shear deformation mode. In the above experiments, only the actuated muscle modules are in flexible state, while the other skeleton/muscle modules remain in rigid states. Notably, softening the nonactuated muscle module could markedly change the effective stiffness distribution, leading to distinct dynamic responses. For example, the actuation of muscle module M2 results in different levels of bending amplitudes, when M1 is in rigid and soft states (fig. S18).

These examples serve as actuator-level demonstrations of distinct musculoskeletal designs. The peacock-like and woodpecker-like actuators emphasize morphology-motion coupling enabled by a single muscle module, and the scorpion-like actuator highlights the tunability of vibration amplitude through stiffness redistribution in a more complex serial layout. Moreover, the human-shaped actuator (Fig. 1E), which integrates multiple muscle and skeleton modules within one platform, provides a comprehensive demonstration of the actuator characteristics, including complex dynamic behaviors and static load-bearing capability.

### Morphable small-scale robot with quadruped and humanoid locomotion modes

The independent programmability of morphology and dynamic behavior, together with a lightweight yet load-bearing structural design, enables the proposed modular musculoskeletal concept to be extended from isolated actuators to fully integrated robotic systems. Realizing, these small-scale robots further requires addressing several system-level design considerations, including appropriate mass distribution to maintain stability during locomotion and shape transformation, optimization of electrical interconnection layout to avoid constraint on the motion, and the incorporation of anisotropic friction to enable effective ground movements. Figure 4 and movie S6 present a morphable small-scale robot (weight: 1.8 g) that can be reversibly transformed between quadruped (7 cm by 3 cm by 1.2 cm) and humanoid states (5.2 cm by 3 cm by 5.5 cm). As shown in Fig. 4A and fig. S19, the robot is composed of a body (skeleton module), a supporting leg (skeleton module), and two pairs of active legs (front and rear; integrated muscle and skeleton modules). Each muscle module incorporates four stacked PDMS layers and corresponding flexible electrodes. Coordinated deformation of the skeleton modules within the front and rear legs enabled the robot to transition from the initial planar state to the quadruped state, analogous to quadruped animals rising from a prone resting posture (Fig. 4A). By lowering the supporting leg to stabilize the body and subsequently folding the top skeleton module to raise the upper body, the robot was further transformed into the humanoid state. This morphological change freed the front legs from the locomotion function, allowing them to serve as arms for manipulation tasks.

The quadruped locomotion is achieved by alternating actuation of the two front and two rear muscles, as shown in Fig. 4B and fig. S20. Figure 4C shows a representative quadrupedal locomotion process, including fast locomotion (1.7 BL/s) on the ground, body lowering through leg deformation, and passing through a low-height channel (5 mm) with an average speed of 0.19 BL/s. The relationship between leg bending angle and voltage applied to LCE layers within the leg skeleton modules is shown in Fig. 4D, providing guidance for the posture control. As *U*_SMP-M_ increases from 0 to 6 V, the muscle stiffness decreases, resulting in a reduction in the resonant frequency from 47 to 9 Hz, while the locomotion speed exhibits a trend with an initial increase followed by a decrease (Fig. 4E). The robot achieved its maximum relative quadruped speed (3.0 BL/s) at *U*_SMP-M_ = 4.5 V under 11.5-Hz resonant excitation (Fig. 4, E and F). To improve the load-bearing capacity, a relatively high muscle stiffness was adopted with use of *U*_SMP-M_ = 2 V, enabling a moderate locomotion speed of 0.30 BL/s while carrying a 6 g weight (>3 times higher than the robot weight) (Fig. 4F). The humanoid locomotion is realized by alternating actuation of the two rear muscle modules (Fig. 4G and fig. S20). Note that the folding angle of the top skeleton module can be regulated by the voltage applied to the LCE layer (Fig. 4H). Figure 4I and movie S6 show the humanoid locomotion while carrying a black plastic tube using the first two arms. To further enhance the manipulation capability, an additional gripper skeleton was integrated with the left front arm. Figure 4J and fig. S21 demonstrate the capability of the robot enriched by such modified design, including bending down to grasp a paper ball, straightening the body, and swinging the arm while opening the gripper to release the ball.

### Sugar glider–inspired untethered multimodal small-scale robot

**Fig. 5.**
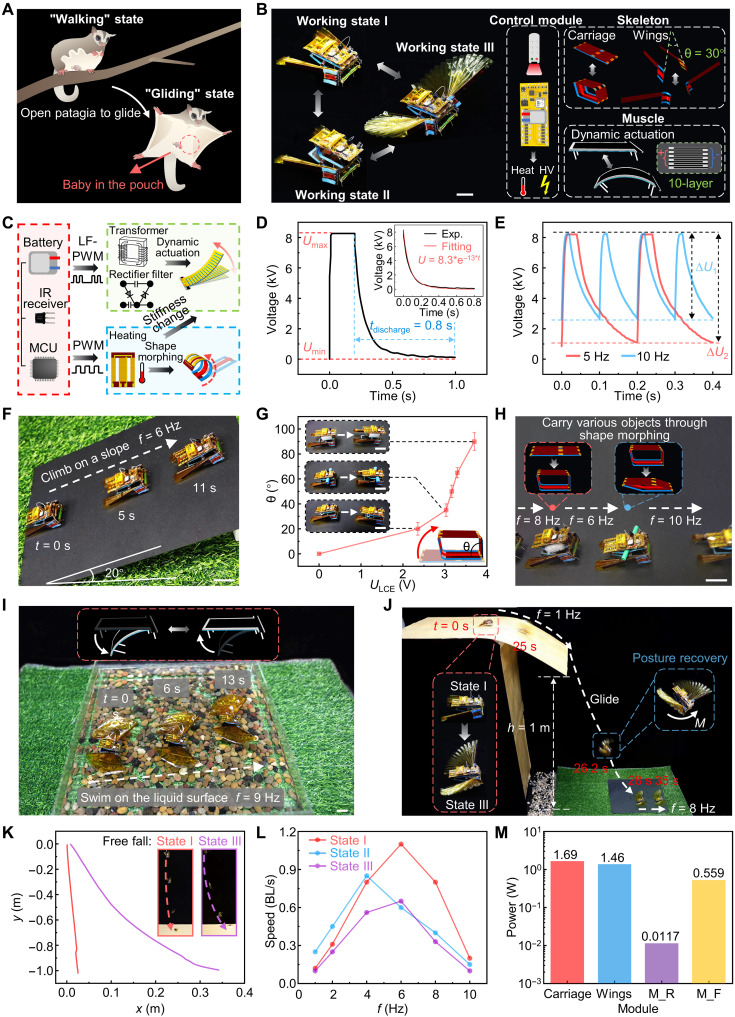
Sugar glider–inspired untethered multimodal small-scale robot. (**A**) Illustration of typical capabilities of a sugar glider, including walking locomotion, gliding from elevated branches, and carrying a baby in its pouch. (**B**) Design and illustration of the sugar glider-inspired robot. The left panel shows optical images of three representative working states. The right panel shows key components of the robot, including the control module, skeleton modules, and muscle module. Scale bar, 2 cm. (**C**) Simplified block diagram of the HV power supply, control components, and their connections to muscle and skeleton modules. (**D**) HV output of the ultralight onboard circuit at 1 Hz, with the inset showing the voltage decay from *U*_max_ to *U*_min_. (**E**) Alternating HV outputs at 5 and 10 Hz. (**F**) Demonstration of the robot climbing a slope with a 20° inclination. Scale bar, 2 cm. (**G**) Carriage opening angle versus the voltage applied to its LCE layer, with insets showing cargo-carrying capability at different opening angles. Scale bars, 2 cm. (**H**) Optical images of the robot carrying cargos of different shapes during terrestrial locomotion. Scale bar, 2 cm. (**I**) Demonstration of liquid-surface swimming. Scale bar, 2 cm. (**J**) Demonstration of multimodal locomotion in a complex scene, in which the robot first morphs into working state III to drive on a high platform, then glides off a cliff, lands stably, and moves forward with terrestrial locomotion. (**K**) Trajectories of the robot during free fall from approximately 1 m in working states I and III. (**L**) Dependence of the terrestrial speed on the actuation frequency in the three working states. (**M**) Energy consumption associated with carriage opening, wings deployment, and muscle actuation at 10 Hz in rigid (M_R, *U*_SMP-M_ = 0 V) and flexible (M_F, *U*_SMP-M_ = 3.7 V) states. Photo credit: S.X., Tsinghua University.

Inspired by the sugar glider, which is capable of walking locomotion, gliding via deployable patagia, and carrying a baby in its pouch, we developed an untethered multimodal small-scale robot (5 cm by 2 cm by 1.5 cm in the undeployed state; 6.4 g) based on the musculoskeletal actuators with a lightweight control module (Fig. 5, A and B, and fig. S22). The muscle module consists of a 10-layer PDMS-based DE, an SMP layer (200 μm), and a Cu/PI (2/15 μm) bilayer, while four attached fine metal pins provide anisotropic friction during terrestrial locomotion. The skeleton modules, including a morphable carriage and a pair of deployable wings, enable the shape transformation among three representative working states (states I to III) (Fig. 5B). Specifically, state I allows fast terrestrial locomotion, state II corresponds to the cargo-carrying mode with an artificial pouch, and state III enables gliding and liquid-surface swimming by leveraging the deployed wings (movie S7). In addition to the above modules, the control module includes an ultralight control circuit (4 cm by 2 cm; 1.04 g) and a lightweight lithium battery (3.7 V, 50 mAh; 1.12 g).

The development of a lightweight onboard kilovolt-level alternating-current power system has been a grand technical challenge for untethered control of DE-based small-scale robots. In this study, the onboard control circuit uses a strategy of low-frequency pulse-width modulation (LF-PWM) to regulate the charging and discharging phases of the HV output, which allows generation of alternating HV waveforms with peak voltages of up to ∼9.5 kV (Fig. 5C, fig. S23, and movie S7). Besides, the PWM can also regulate the effective voltage level applied to the heating electrodes, enabling the control of muscle stiffness and skeleton deformation (fig. S24). Owing to the approximately quadratic dependence of muscle deformation amplitude on the applied HV (fig. S7), variations in the low-voltage level have a limited influence on the deformation amplitude. By exploiting the fast charging and slow discharging characteristics of the onboard circuit (Fig. 5D), we achieved low-to-mid-frequency HV actuation (e.g., 1 to 10 Hz) through coordinate regulation of the low-voltage (*U*_min_) level and high-voltage (*U*_max_) holding time. Notably, the breakdown voltage of the DE in this robot is ∼8.5 kV. Figure 5E, fig. S25, and movie S7 demonstrate alternating HV outputs at 5 and 10 Hz, with the low-voltage level increasing as the actuation frequency rises. Using an infrared (IR) remote controller shown in fig. S26, the shape transformation of the skeleton modules, the stiffness regulation of the muscle module, and the actuation frequency control (1 to 10 Hz) can be independently programmed. Higher actuation frequencies can be realized by increasing the low-voltage level to accelerate the discharge process, although this leads to an increased power consumption and a reduced achievable actuation amplitude.

The terrestrial locomotion of the robot at the undeployed state (state I) under different frequencies (1 to 10 Hz) is shown in movie S8, and Fig. 5F demonstrates the climbing on a slope with a 20° inclination. The controlled transformation of the carriage enables the robot to carry cargos with diverse shapes, such as paper clips, plastic tubes, and foam blocks (Fig. 5G and movie S9). Figure 5H and movie S9 show the robot carrying cargos of different shapes while moving forward, showcasing the powerful capabilities of the artificial pouch (carriage). By increasing muscle stiffness through the applied heating voltage (*U*_SMP-M_), a higher load-bearing capacity is achieved, enabling the robot to carry a 3.6-g silicone block (56% of the robot weight) at *U*_SMP-M_ = 2 V (fig. S27 and movie S9). Owing to the light weight and the large wing area—induced buoyant force, the robot can swim on a viscous liquid surface (silicone oil, 100 cSt) at the wing-expanded state (state III), with an average speed of 0.2 BL/s (Fig. 5I and movie S10). In addition to silicone oil, swimming on the water surface was also conducted, after encapsulating the circuit with a PI coating layer to ensure electrical insulation (fig. S28 and movie S10). In this case, the deformation amplitude of the muscle module decreases, which is likely due to partial water ingress into the multilayer PDMS and enhanced convective cooling that suppresses the softening of the SMP layer. Nevertheless, the robot still exhibits rapid surface locomotion speed (0.37 BL/s at 10 Hz) because the lower viscosity of water (≈1 cSt at 25°C) allows efficient propulsion even with a reduced actuation amplitude. Notably, the vibration mode of muscle module during terrestrial locomotion resembles that of a beam with free ends, while the vibration mode during swimming is closer to that of a cantilever beam. When inclined upward, the deployable wings can also provide sufficient lift during the gliding process (fig. S29). As shown in Fig. 5J and movie S10, the robot first morphed into the wing-expanded state to drive on a high platform, then glides off a cliff, and lasty lands to continue terrestrial locomotion. Comparative free-fall tests in states I and III were carried out, further proving that the lift generated by the wings evidently improved posture recovery and landing stability (Fig. 5K and movie S10). The relation between relative terrestrial speed and actuation frequency for the three working states is shown in Fig. 5L, indicating that the robot offers the highest terrestrial speed (1.1 BL/s) at the undeployed state. The energy consumptions associated with carriage opening, wings deployment, and muscle actuation at 10 Hz in rigid (*U*_SMP-M_ = 0 V) and flexible (*U*_SMP-M_ = 3.7 V) states are demonstrated in Fig. 5M and figs. S30 to S32. The measurements for muscle were conducted at 10 Hz, where the power consumption is relatively high. The energy cost for shape transformation remains below 3.5% of the battery capacity (3.7 V, 50 mAh). In addition, the peak power consumption of 0.559 W for the muscle corresponds to a continuous operation time of ∼20 min under the worst-case condition, noticing that the flexible muscle state additionally accounts for electrothermal power required for stiffness modulation, thereby providing a conservative assessment. In general, reducing muscle stiffness, increasing actuation frequency, and increasing the DE driving voltage all lead to higher energy consumption in the present robotic system. From a design perspective, increasing muscle stiffness to a certain extent can improve payload capability while reducing electrothermal energy consumption. In contrast, higher locomotion speed typically requires lower muscle stiffness to allow larger deformation amplitude, which increases electrothermal energy consumption. Further speed enhancement can be achieved by a moderate increase in actuation frequency, but this also leads to a higher electrical energy consumption of DE actuation.

Compared with other representative untethered morphable robots with onboard power systems (*8*, *9*, *11*, *12*, *18*), the present robot, whose actuation system is entirely composed of soft materials, achieves the smallest size and lightest weight while offering a relatively high level of functional complexity (table S1). The proposed musculoskeletal actuators with programmable morphology and tunable dynamics thus provide an effective pathway toward miniaturized multimodal robots without sacrificing the capability of shape transformation or agile locomotion.

## DISCUSSION

This work presents a class of artificial musculoskeletal actuators constructed with serially connected morphable skeleton modules and stiffness-tunable muscle modules, which enable independent programming of morphology and dynamic behavior. These capabilities allow developments of geometrically sophisticated musculoskeletal actuators that can reproduce complex dynamic behavior of natural species (e.g., woodpecker and scorpion). A centimeter-scale morphable robot with both quadruped and humanoid locomotion modes is demonstrated, which is difficult to achieve at similar sizes. Moreover, the integration of customized musculoskeletal actuators with a lightweight control module enables the realization of an untethered sugar glider-inspired small-scale robot (5 cm by 2 cm by 1.5 cm in the undeployed state; 6.4 g), exhibiting fast terrestrial locomotion (e.g., 1.1 BL/s), cargo carrying (e.g., 3.6 g), liquid-surface swimming (e.g., 0.37 BL/s), and aerial gliding. Together, these results suggest that coordinating structural transformation with dynamically tunable actuation can serve as an effective strategy for multimodal robots.

Future work could focus on improving the long-term reliability and the electrical insulation of the DE layer to extend operational lifetime, as well as optimizing the onboard circuit to expand the accessible actuation frequency range without sacrificing the actuation amplitude. Lightweight onboard sensors—such as inertial, optical, or pressure sensors—could also be integrated for real-time monitoring of robot state and surrounding environment, enabling the microcontroller to regulate skeleton reconfiguration, muscle stiffness, and actuation frequency. These sensing and feedback-based closed-loop control modules (*52*–*58*) could enhance robustness and adaptability of the untethered robot in unstructured environments, addressing practical demands. Furthermore, the proposed ultralight onboard control circuit could be extended to robotic systems using HASEL or other electrostatic actuators (*59*). The musculoskeletal actuators may also find applications in wearable haptic devices requiring complex and programmable dynamic responses (*60*–*62*).

## MATERIALS AND METHODS

### Preparation of SMPs and LCEs for the musculoskeletal actuator

SMP films were synthesized by mixing epoxy monomer E44 (molecular weight ≈ 450 g mol^−1^; China Petrochemical Corporation, China) with the curing agent JEFFAMINE D230 (molecular weight ≈ 230 g mol^−1^; Sigma-Aldrich, China). The mixture was degassed in a vacuum drying oven (Tianjin Gongxing, China) and then poured into glass cells with gaps of specific sizes. Subsequent curing of the mixed solution in a furnace (110°C) for 1 hour allowed formation of SMP films. A CO_2_ laser cutting machine (VLS2.30, USA) enabled formation of customized patterns. Adjusting the ratio of E44 and D230 during the above process provides a means to tune the mechanical and thermal properties of the resulting SMP films. More details about the compositions of SMPs are available in table S2.

The main-chain nematic LCE with diacrylate mesogen (RM257) was synthesized using two-stage thiol-acrylate Michael addition and photopolymerization reaction. Specifically, 1,4-bis-[4-(3-acryloyloxypropyloxy) benzoyloxy]-2-methylbenzene (RM257, Shijiazhuang Yesheng Chemical Technology, China) and (2-hydroxyethoxy)-2-methylpropiophenone (Energy Chemical, China) were dissolved in toluene at 80°C. In another beaker, 2,20-(ethylenedioxy) diethanethiol (Energy Chemical, China), pentaerythritol tetrakis (3-mercaptopropionate) (Energy Chemical, China), and dipropyl amine (Energy Chemical, China) were dissolved in toluene at room temperature. These two solutions were then mixed and poured into a borosilicate glass dish, followed by degassing in a vacuum drying oven (Tianjin Gongxing, China) to remove air bubbles. The mixture was cured overnight at room temperature to form a slightly crosslinked polydomain LCE film. Before integration into the actuator, a uniaxial prestretch and a subsequent ultraviolet (UV) exposure enabled the second-stage polymerization reaction of LCE film. More details about the compositions of LCEs are available in table S3.

### Characterization of SMPs

The dynamic mechanical analysis (DMA) of SMP samples was conducted using a DMA Q850 system (TA Instruments, USA) (fig. S4B), where the experimental conditions were tensile, “multifrequency, strain” mode at 1 Hz, 0.2% strain, and a heating rate of 3°C min^−1^. The uniaxial stress-strain curves of SMP at different temperatures were obtained from DMA measurements, from which the elastic moduli were extracted (fig. S4C).

### Preparation of the muscle module

The fabrication process of the muscle module (fig. S3) involves the preparation of a multilayer PDMS-based DE and its integration with an SMP layer. Acrylic circular frames were fabricated by laser cutting to support PDMS membranes (100 μm for each layer), and then carbon grease (NyoGel 756 G, Nye Lubricants, USA) electrodes were patterned on both surfaces of PDMS membranes using shadow masks, yielding single-layer DE units. The uniformity of electrode coating was carefully controlled to ensure stable deformation and high dielectric breakdown strength. Sequential UV-ozone treatment, conformal lamination, and thermal treatment were adopted for the multilayer PDMS to enable strong interlayer bonding via siloxane linkages. After completing the multilayer structure, a Cu/PI (2/15 μm) bilayer kirigami heating electrode fabricated by lithography and an SMP layer (150 μm) were laminated onto the actuator using an ultrathin silicone adhesive room temperature vulcanising glue (NAN DA 705, Liyang Kangda Chemical Company, China) to minimize thickness and preserve mechanical stability. The final geometry of the muscle module was defined by laser cutting, and electrical wires were connected separately to the DE electrodes and the heating electrode before operation.

### Finite element analyses

The commercial software ABAQUS (SIMULIA) was used to perform the FEAs on the deformations of the actuator. An implicit solver was adopted to calculate deformed configurations of the actuator, with the geometric nonlinearity considered. For the muscle module, shell elements (S4R) were used for PI, and solid elements (C3D8RH) were used for PDMS and SMP. A material model reported in a previous study (*63*) was embedded into the software with the user-defined subroutine UMAT to simulate the actuation of PDMS. For the skeleton module, shell elements (S4R) were used for PI, and solid elements (C3D8RH) were used for LCE and SMP. Refined meshes ensured the computational accuracy. Linear, elastic constitutive relations were used for PI, SMP, and LCE for simplicity. A temperature-dependent expansion was exploited to model the nonlinear thermal actuation responses of LCE.

### Characterization and actuation of the muscle module

The electrothermal operation was controlled by dc power supplies (TD1326, TASI, China). The DE layer was powered by a high-voltage source, which was generated by a signal generator (DG4102, Rigol, China) and a high-voltage amplifier (610E, TREK, USA). An IR camera (VarioCAM HD head, InfraTec, Germany) and a digital camera (760D, Canon, Japan) recorded thermal and optical mapping images/videos of actuators under different applied voltages. The static deformation was measured on the basis of optical images, while dynamic deformation amplitudes under different driving frequencies were characterized using a laser Doppler vibrometer system (Polytec GmbH, Germany, fig. S11). Also, the force-distance curves (fig. S8) during bending deformations were used to characterize the equivalent bending stiffness (*EI*) based on the following formula

$$\text{EI}=\frac{{\text{FL}}_{0}^{3}}{3d}=\frac{{\mathrm{kL}}_{0}^{3}}{3}$$ (1)

where *d* and *F* are the distance and the force applied to the free end of the muscle skeleton, *L*_0_ is the muscle length, and *k* is the slope of the force-distance curve. Besides, the actuation force of the muscle module was characterized using the experimental setup shown in fig. S11.

### Onboard control circuit design

The onboard control circuit was designed to provide high-voltage actuation for musculoskeletal modules while maintaining a compact size and an ultralight weight (fig. S23). The circuit adopts a forward-flyback hybrid topology combined with a multistage Cockcroft-Walton voltage multiplier to achieve kilovolt-level voltage output amplified from a low-voltage battery input (3.7 V) using a single switching device (*64*). By operating the MOSFET near resonance, the low-voltage dc input is converted into an AC signal, amplified through magnetic coupling, and further boosted by the voltage multiplier to generate high-voltage outputs of up to ∼9.5 kV. To enable programmable actuation rather than static high-voltage output, a PWM is used to regulate the effective HV level. In addition, a LF-PWM strategy is implemented to modulate the charging and discharging phases of the HV output, enabling the generation of low-to-mid-frequency alternating HV waveforms. Besides, the PWM can also regulate the effective voltage level applied to the heating electrodes, allowing the control of muscle stiffness and skeleton deformation. The wireless control is achieved by integrating an IR receiver into the onboard circuit. Commands from an external IR remote controller are decoded by the microcontroller to adjust PWM duty cycles and LF-PWM parameters in real time, enabling remote switching between different stiffness states, skeleton deformation modes, and muscle actuation frequencies.

### Onboard circuit encapsulation for water-surface swimming

To enable safe operation of the untethered robot on the water surface, the circuit was immersed in PI solution to form a conformal coating. After removal from the solution, the coated circuit was thermally cured in an oven (100°C) to evaporate the solvent and form a dense insulating layer on the circuit surface. PI was selected because of its high dielectric strength, low water absorption, and good thermal stability, which are advantageous for insulating high-voltage electronics in liquid environments while adding relatively small weight to the robot.

### The untethered multimodal small-scale robot

The robot consists of a lithium battery (3.7 V and 50 mAh), an ultralight control circuit (1.04 g), two skeleton modules (carriage and wings), and a muscle module. The fabrication began with the preparation of customized skeletons. Sequential folding and thermoforming of a thin PI film (25 μm), followed by adhering with the wing skeleton (LCE, 800 μm; SMP, 500 μm), completed the preparation of the morphable wings. The body (muscle module) was fabricated by laminating 10-layer PDMS (total thickness: 1 mm), flexible electrodes (carbon grease; NyoGel 756 G, Nye Lubricants, USA), an SMP layer (200 μm), and a Cu/PI (2/15 μm) layer. Then, the superglue (Gorilla Glue Company, USA) was used to connect different components.

### Energy cost of the untethered multimodal small-scale robot

The power consumption of the skeleton modules used in the robot can be calculated from the following formulae (Fig. 5M)

$$P_{\text{Skeleton}}=U_{\text{LCE}}I_{\text{LCE}}+U_{\text{SMP}-S}I_{\text{SMP}-S}$$ (2)

where *U*_LCE_ and *U*_SMP-S_ are voltages applied to LCE and SMP ribbons within skeleton modules, respectively; *I*_LCE_ and *I*_SMP-S_ are currents passing through LCE and SMP ribbons, respectively. The power consumption of the DE layer within the muscle module was measured by the setup shown in fig. S31

$$P_{\text{DE}}=\frac{\int_{0}^{T}U_{\text{Muscle}}\frac{U_{\text{R}}}{R}\;dt}{T}$$ (3)

where *U*_Muscle_ and *U*_R_ refer to the voltages measured across the DE and series resistor, respectively, and *R* and *T* refer to resistance value of the series resistor and time period. The power consumption of the muscle module was the sum of the power consumed by the associated DE layer and the heating electrode

$$P_{\text{Muscle}}=P_{\text{DE}}+P_{\text{Electrode}}=P_{\text{DE}}+\frac{{U_{\text{Electrode}}}^{2}}{R_{\text{Electrode}}}$$ (4)

Here, *U*_Electrode_ denotes the voltage applied to the heating electrode within the muscle module, which is adjustable via PWM (fig. S24), and *R*_Electrode_ refers to the electrical resistance of the heating electrode. For the muscle module operating in rigid and flexible states, *U*_Electrode_ was set to 0 and 3.7 V, respectively. Furthermore, the portion of energy consumption for the shape transformation of skeleton modules can be calculated as follows (fig. S32)

$$\eta =\frac{U_{\text{LCE}}I_{\text{LCE}}t_{\text{LCE}}+U_{\text{SMP-S}}I_{\text{SMP}-S}t_{\text{SMP}-S}}{W_{\text{battery}}}$$ (5)

where *W*_battery_ is the total stored energy of the lithium battery (3.7 V and 220 mAh), which can be calculated using the capacity multiplied by its output voltage. Also, *t*_LCE_ and *t*_SMP-S_ refer to the heating time of LCE and SMP within the skeleton module during the deformation.
