# Supplementary material for: Musculoskeletal actuators with programmable morphology and tunable dynamics
Source: Sci Adv. 2026 Jul 29;12(31):eaeg4524. doi: 10.1126/sciadv.aeg4524 (PMC13418923; doi:10.1126/sciadv.aeg4524)
Supplement: Supplementary file 1 — Supplementary Text S1 to S3 Figs. S1 to S32 Tables S1 to S3 Legends for movies S1 to S10 [file sciadv.aeg4524_sm.pdf]

Science Advances
AAAS

Supplementary Materials for

Musculoskeletal actuators with programmable morphology and tunable dynamics

Shiwei Xu *et al.*

Corresponding author: Yihui Zhang, yihuizhang@tsinghua.edu.cn

Sci. Adv. **12**, eaeg4524 (2026)
DOI: 10.1126/sciadv.aeg4524

The PDF file includes:

Supplementary Text S1 to S3
Figs. S1 to S32
Tables S1 to S3
Legends for movies S1 to S10

Other Supplementary Material for this manuscript includes the following:

Movies S1 to S10

Supplementary Text S1. Stiffness tuning of the muscle module

When different voltages are applied to the heating electrode integrated with the SMP layer, the temperature of the SMP can be continuously tuned using the following formula, as characterized in fig. S5A for the design parameters adopted in this study:

$$T_{\text{SMP-M}} = 25 + 2.46U_{\text{SMP-M}} + 0.78U_{\text{SMP-M}}{}^2, \tag{S1}$$

where $T_{\text{SMP-M}}$ and $U_{\text{SMP-M}}$ take the unit of °C and V, respectively. According to fig. S4C, the SMP exhibits distinct elastic moduli at different temperatures, leading to an evident change in the effective bending stiffness of the muscle module. According to classical vibration theory of cantilever beams, variations in bending stiffness directly result in corresponding changes in dynamic responses, including resonant frequency, vibration amplitude, and actuation force. Therefore, by electrically tuning the SMP stiffness, the dynamic response of the muscle module can be actively regulated.

Supplementary Text S2. Shape morphing and locking of the muscle module

As shown in fig. S9A, when the SMP is heated above its glass transition temperature (T_g) by applying voltage to the heating electrode, its modulus drops sharply, facilitating the deformation of the muscle module (the actuated state) under a DC HV applied to the DE. Subsequently, the heating voltage applied to the SMP is turned off, allowing the SMP to cool and recover its high stiffness. After stiffness recovery, the HV supply to the DE is removed, resulting in a stable, locked deformed configuration (the latched state).

Supplementary Text S3. Shape transformation of the skeleton

Fig. S15 and movie S3 show the time histories of the voltages applied to LCE and SMP ribbons within skeleton modules, and IR/optical images at representative states. During 0-13 s, LCE and SMP ribbons were heated with applied voltages of 1.5 V and 3 V, respectively. The voltage applied to the SMP ribbons was then removed, followed by removal of the voltage applied to LCE ribbon at 19 s, enabling locking of the deformed configuration. To be noted, the molecules in LCE were frozen due to the high stiffness of SMP at room temperature. By adjusting the voltage applied to LCE, the temperature of LCE ribbon (fig. S5B) can be tuned according to:

$$T_{\text{LCE}} = 25 - 0.96U_{\text{LCE}} + 7.73U_{\text{LCE}}{}^2. \tag{S2}$$

For the design studied herein, T_{LCE} and U_{LCE} take the unit of °C and V, respectively. Guided by this relationship, different deformed configurations can be easily achieved and subsequently fixed after cooling of the SMP ribbons.

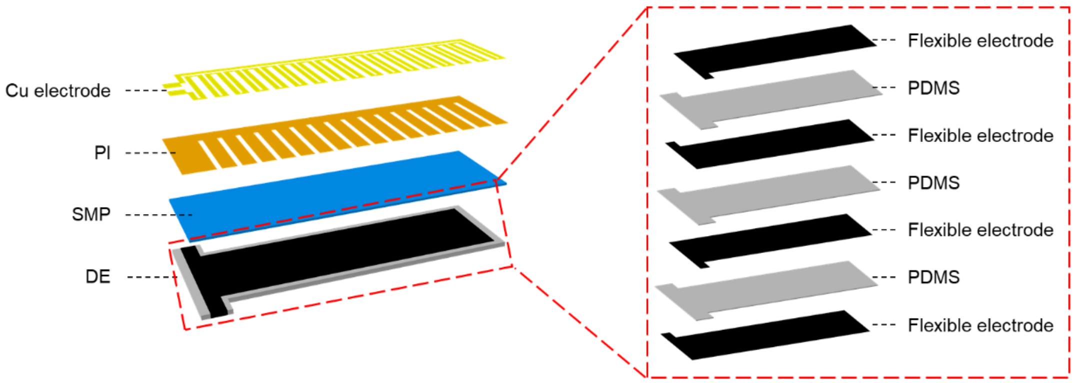

Fig. S1. Exploded view of the muscle module, in which the DE layer comprises multilayer PDMS films and laminated flexible electrodes.

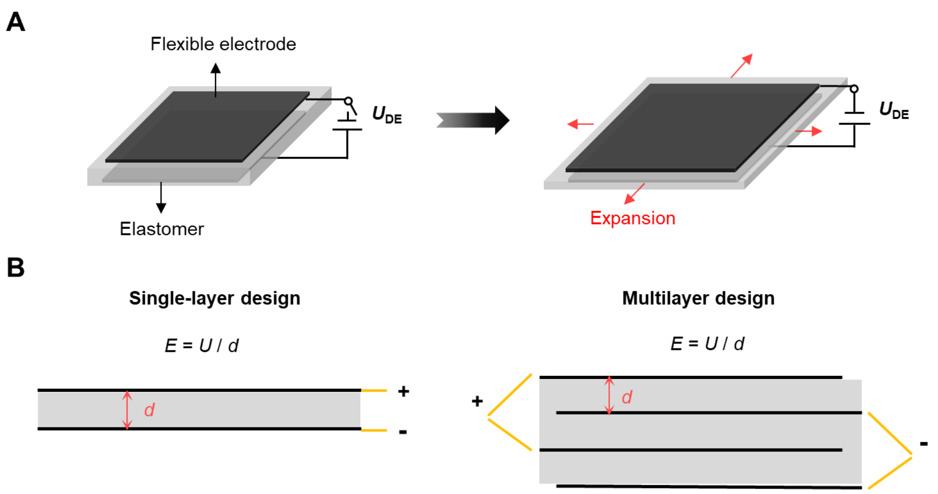

Fig. S2. Schematic illustration of the DE. (**A**) Illustration of DE deformation upon application of a high voltage to the flexible electrodes. (**B**) Comparison of the single-layer and multilayer designs, showing that the multilayer configuration enables the same electric field intensity (E).

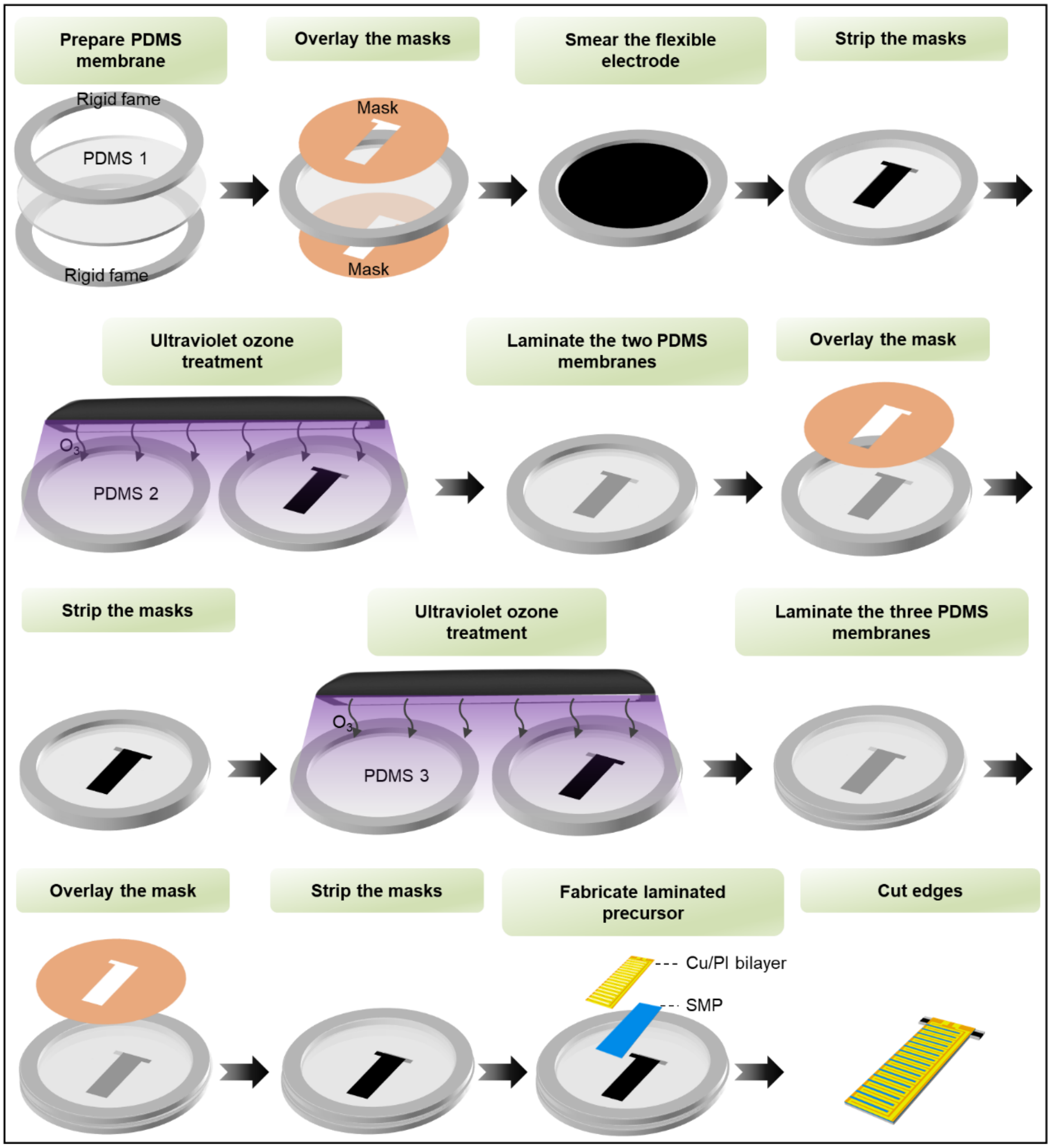

Fig. S3. Schematic illustration of the fabrication process of the muscle module where the DE layer adopts a tri-layer design of PDMS films.

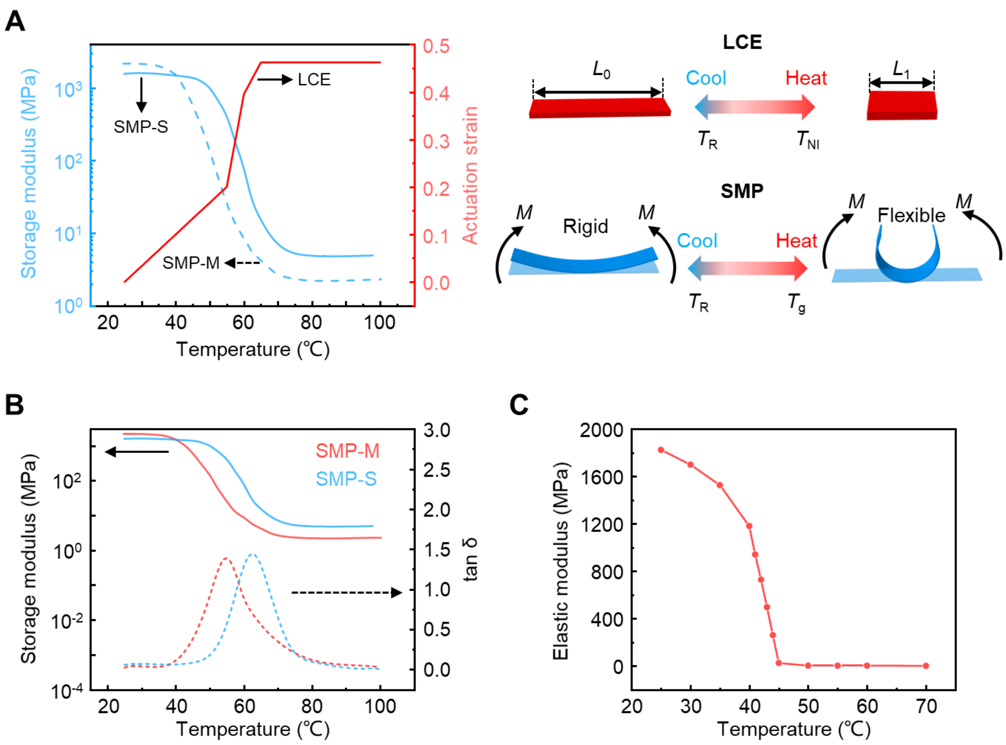

Fig. S4. Design and characterization of the LCEs and the SMPs used in the musculoskeletal actuator. (**A**) Thermal responsive characteristics of LCEs and SMPs. (**B**) Results of dynamic mechanical analysis (DMA) for two types of SMPs. (**C**) Elastic modulus of SMP-M as a function of temperature.

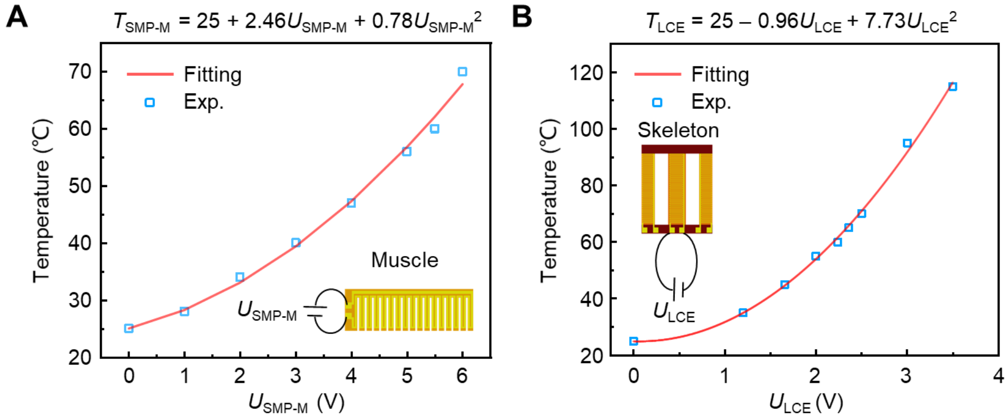

Fig. S5. Characterization of the heating electrodes. (**A**) Relationship between the temperature and the voltage applied to the SMP layer within the muscle module. (**B**) Relationship between the temperature and the voltage applied to the LCE layer within the skeleton module.

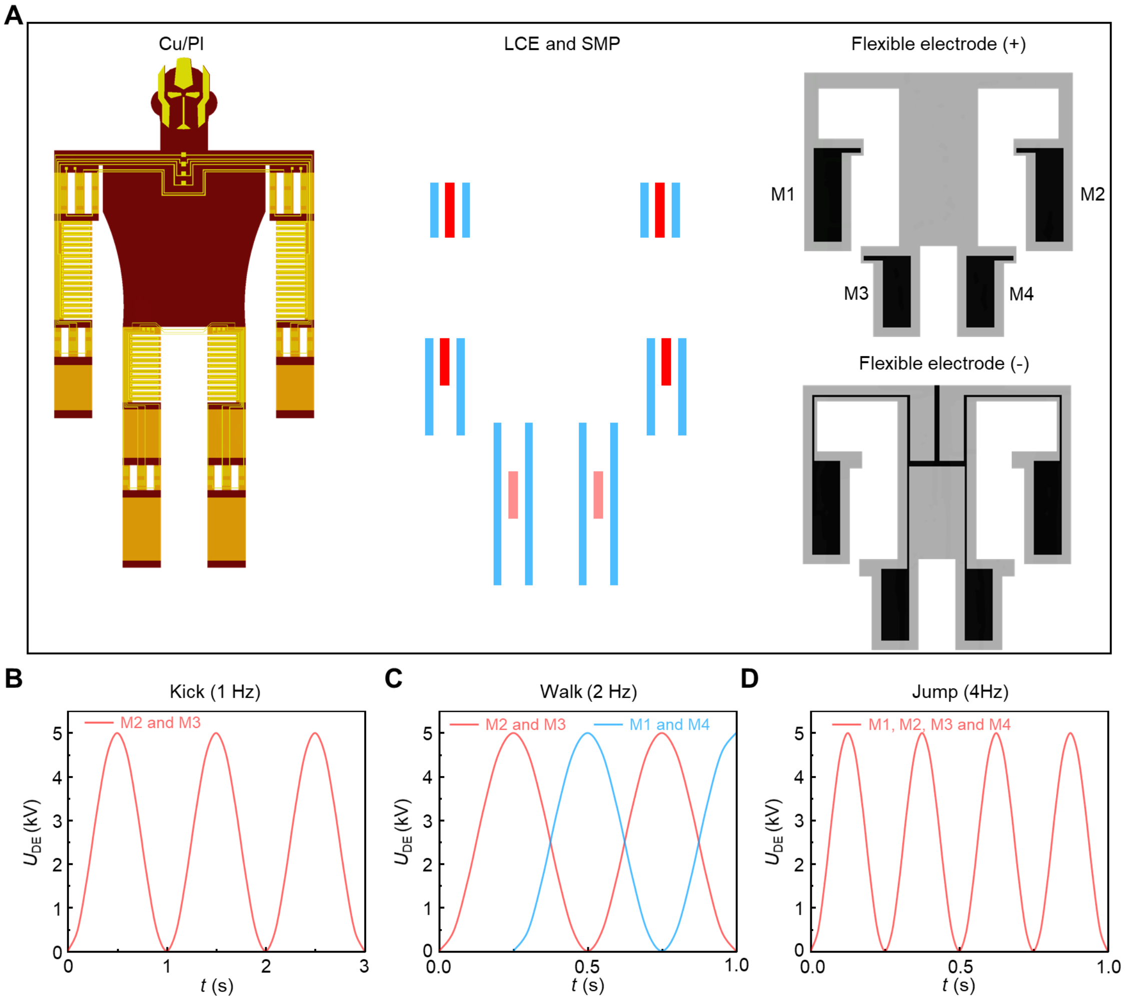

Fig. S6. Design of the human-shaped musculoskeletal actuator. (**A**) Cu/PI pattern, LCE and SMP pattern, and the DE pattern. (**B-D**) Control voltage signals to activate three different types of actions (kick, walk and jump).

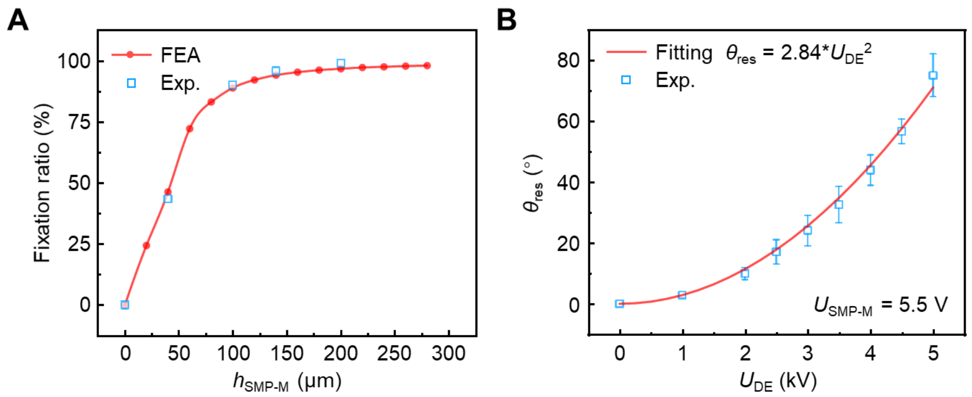

Fig. S7. Design and characterization of the muscle module. (**A**) Shape fixation ratio (defined as ($\theta_{\text{actuated}} - \theta_{\text{latched}}$) / θ_{actuated}) plotted as a function of the SMP layer thickness $h_{\text{SMP-M}}$. (**B**) Static bending angle plotted as a function of the applied voltage U_{DE}, indicating an approximately quadratic relationship. Data are presented as mean values, and error bars are s.d. values from three independent samples.

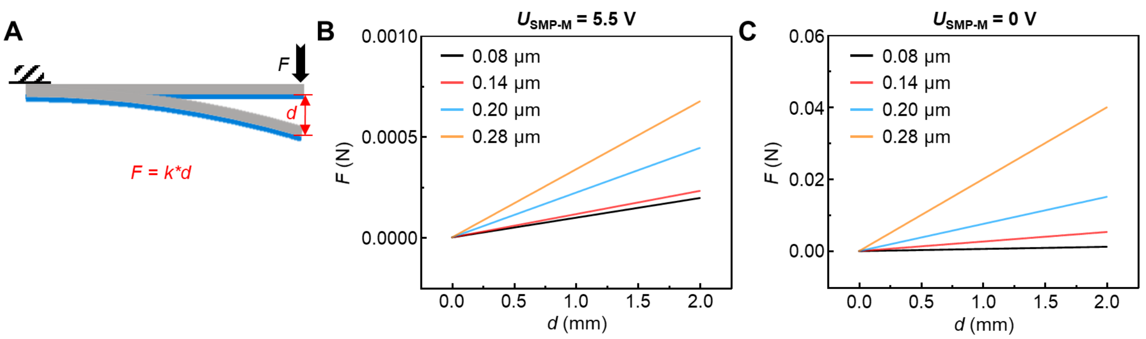

Fig. S8. Bending stiffness test of the muscle module. (**A**) Schematic illustration of the bending test. (**B**-**C**) Load-displacement curves during the bending tests of muscle modules with different SMP layer thicknesses, in both cases of flexible state (**B**) (i.e., $U_{\mathrm{SMP\text{-}M}}$ = 5.5 V) and rigid state (**C**) (i.e., $U_{\mathrm{SMP\text{-}M}}$ = 0 V).

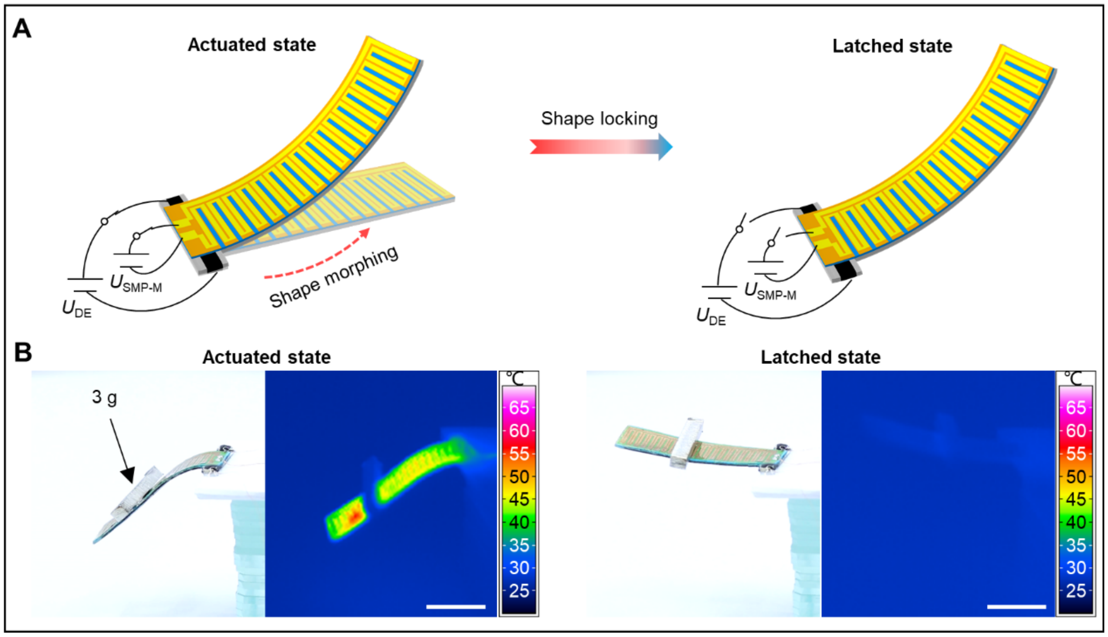

Fig. S9. Demonstration of the shape morphing and locking capability of the muscle module. (**A**) Illustration of the shape morphing and subsequent locking process under DC HV applied to the DE layer. (**B**) Optical and IR images of the muscle module loaded with a 3 g weight in the actuated and latched states. Scale bars, 5 mm.

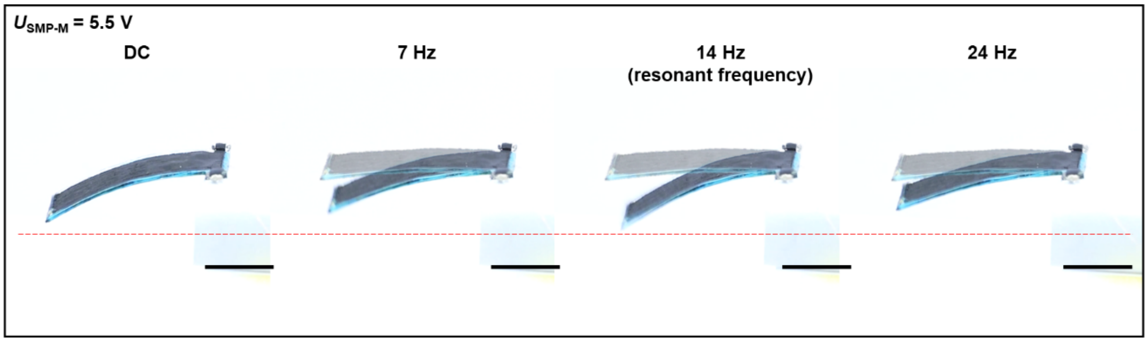

Fig. S10. Demonstration of muscle deformation under different actuation frequencies. Scale bars, 5 mm.

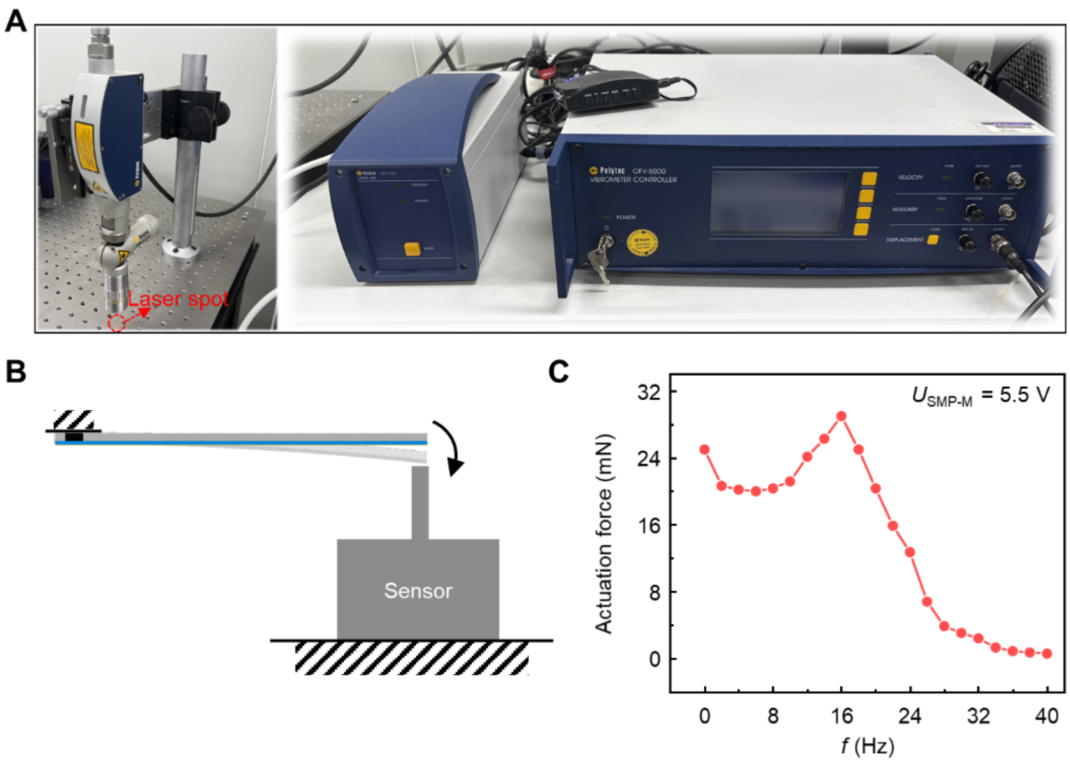

Fig. S11. Characterization of the dynamic deformation and actuation force of the muscle module. (**A**) Optical images of the experimental setup used to measure the dynamic deformation. (**B**) Schematic illustration of the setup used to measure actuation force. (**C**) Influence of the actuation frequency on the actuation force.

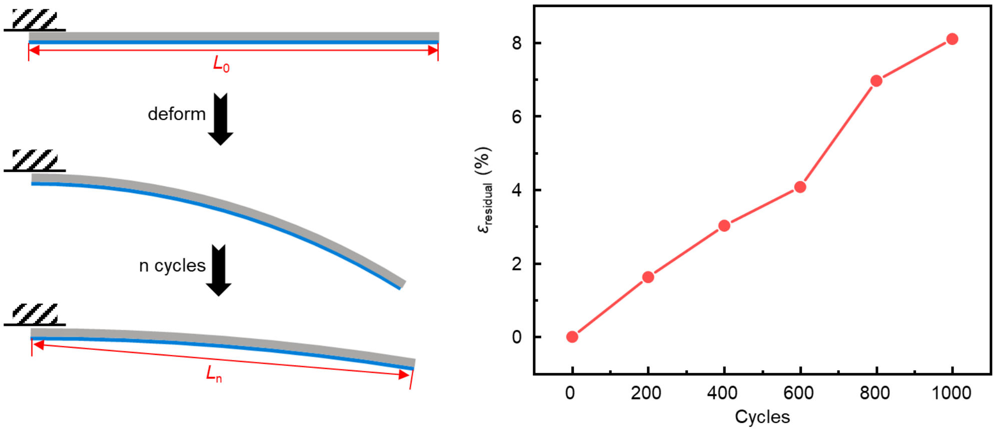

Fig. S12. Measured residual strain ($\varepsilon_{residual} = (L_0 - L_n)/L_0$) versus the number of actuation cycles.

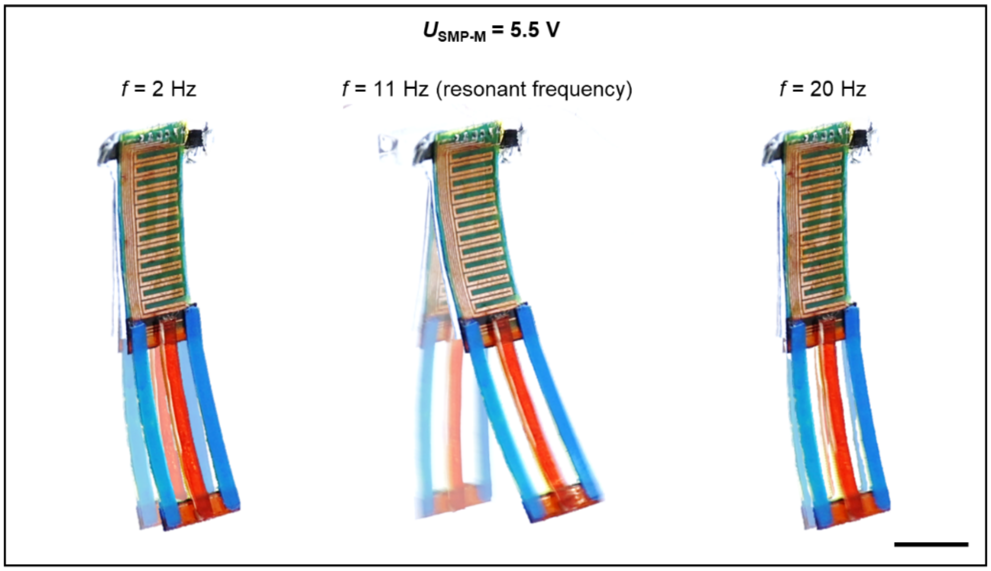

Fig. S13. Optical images of the basic musculoskeletal actuator under different actuation frequencies. Scale bar, 5 mm.

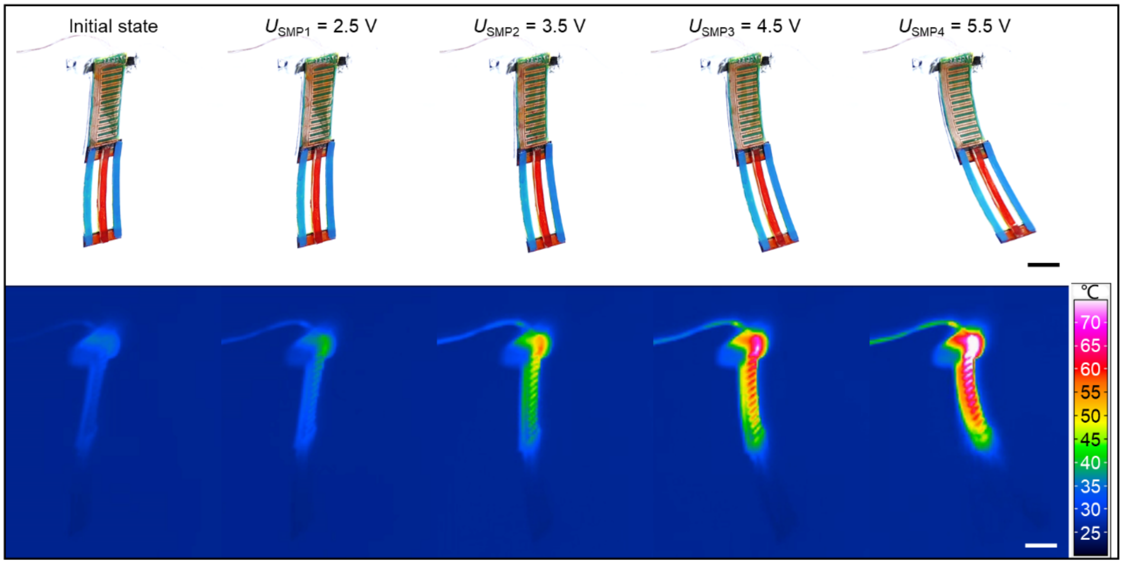

Fig. S14. Optical and IR images demonstrating varied deformations of the basic musculoskeletal actuator under different muscle stiffness levels, for a fixed applied voltage U_{DE} = 5 kV. Scale bars, 5 mm.

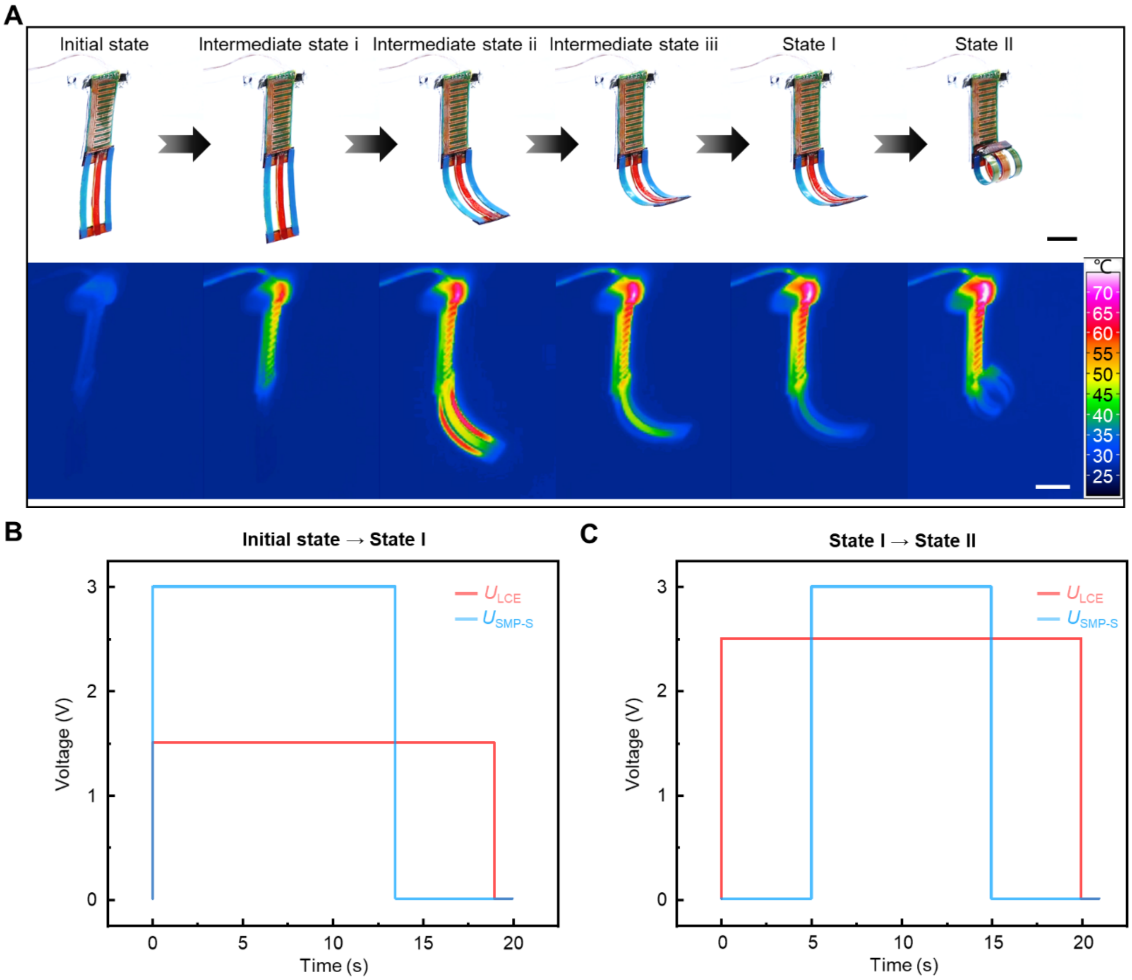

Fig. S15. Demonstration of the typical process shown in movie S1 during actuation of the skeleton module. (**A**) Optical and IR images of the process. Scale bars, 5 mm. (**B**) Time histories of voltage signals applied to LCE and SMP layers within the skeleton module during shape transformation from the initial state to state I. (**C**) Time histories of voltage signals applied to LCE and SMP layers within the skeleton module during shape transformation from state I to state II.

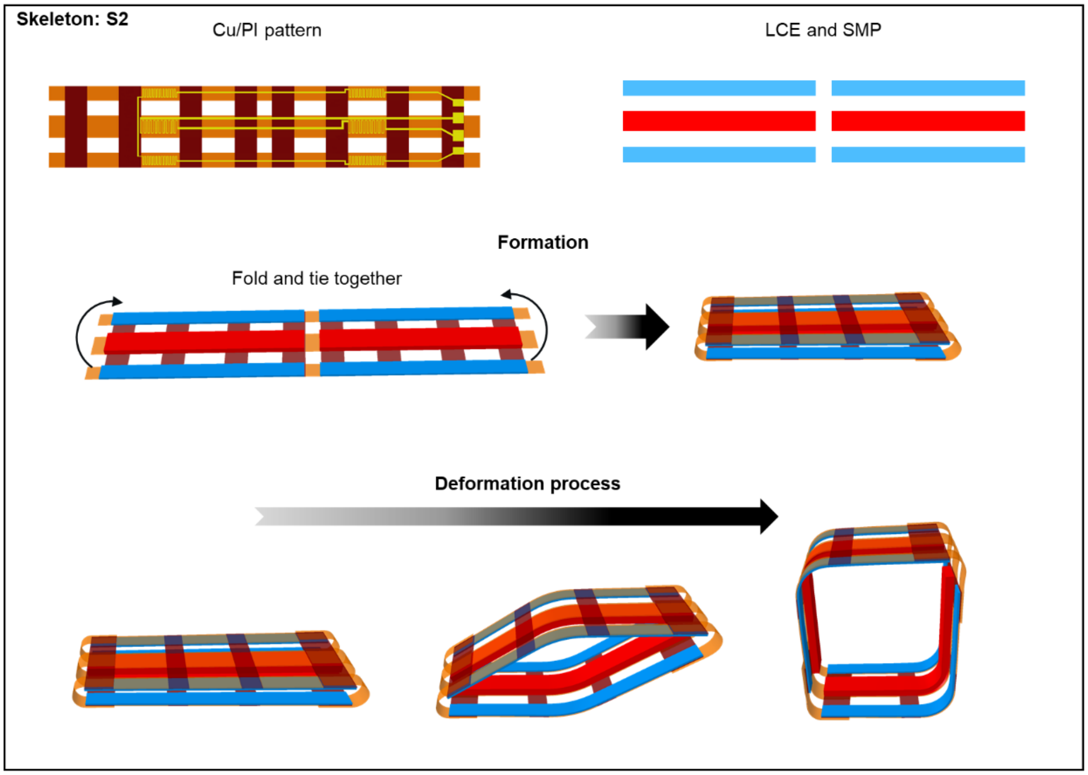

Fig. S16. Cu/PI pattern, LCE and SMP pattern, formation, and deformation process of the skeleton module 'S2' shown in Fig. 3B.

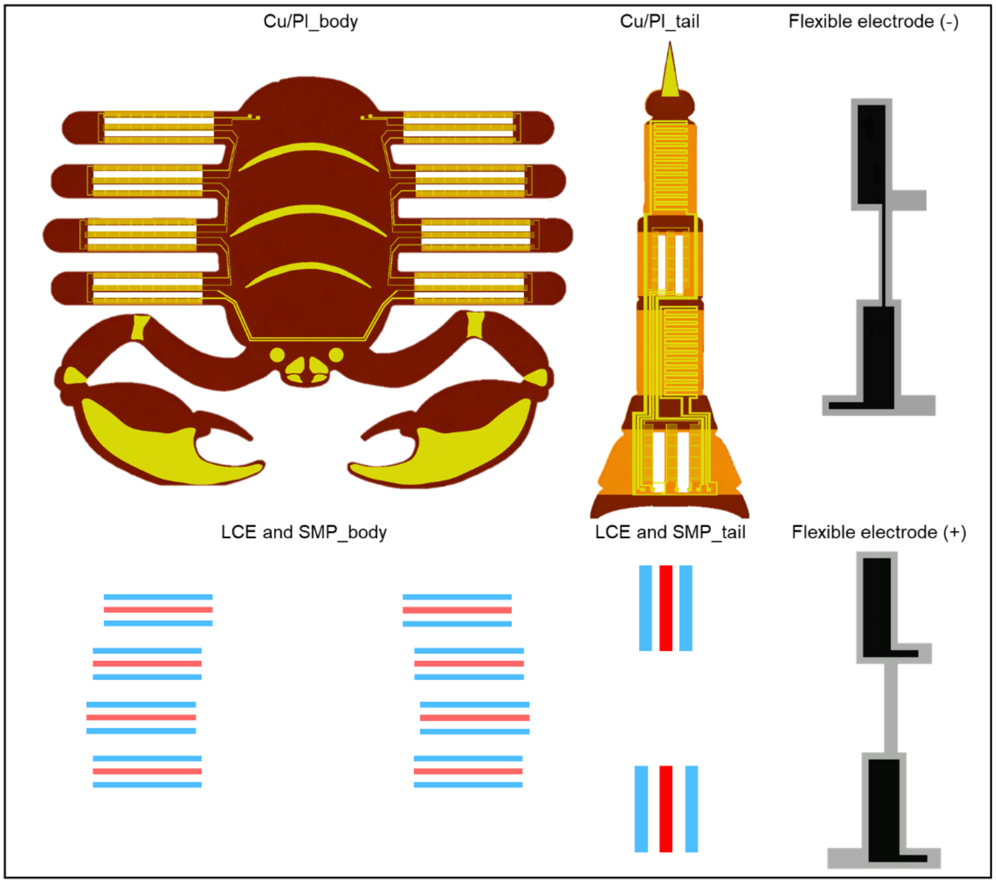

Fig. S17. Cu/PI pattern, LCE and SMP pattern, and DE pattern of the scorpion-like actuator shown in Fig. 3C.

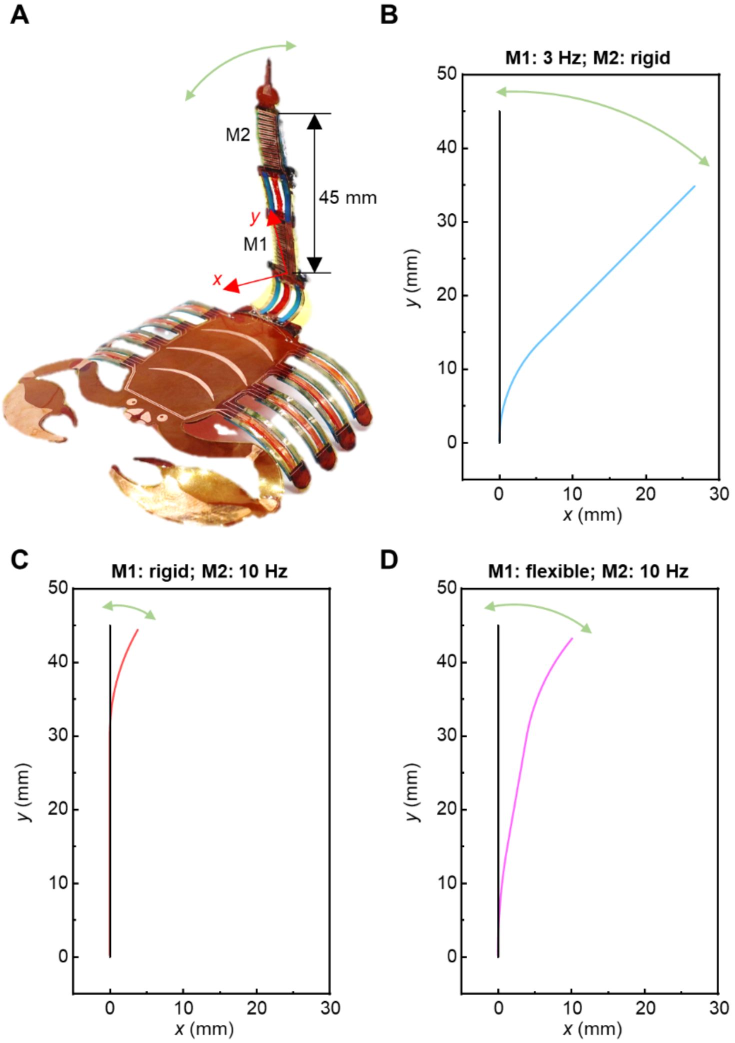

Fig. S18. Tail bending response of the scorpion-like actuator in state II. (**A**) Optical image of the scorpion actuator in state II. (**B**) Bending response with the muscle module M1 actuated while keeping the muscle module M2 rigid. (**C**) Bending response with the muscle module M2 actuated while keeping the muscle module M1 rigid. (**D**) Bending response with the muscle module M2 actuated while keeping the muscle module M1 flexible.

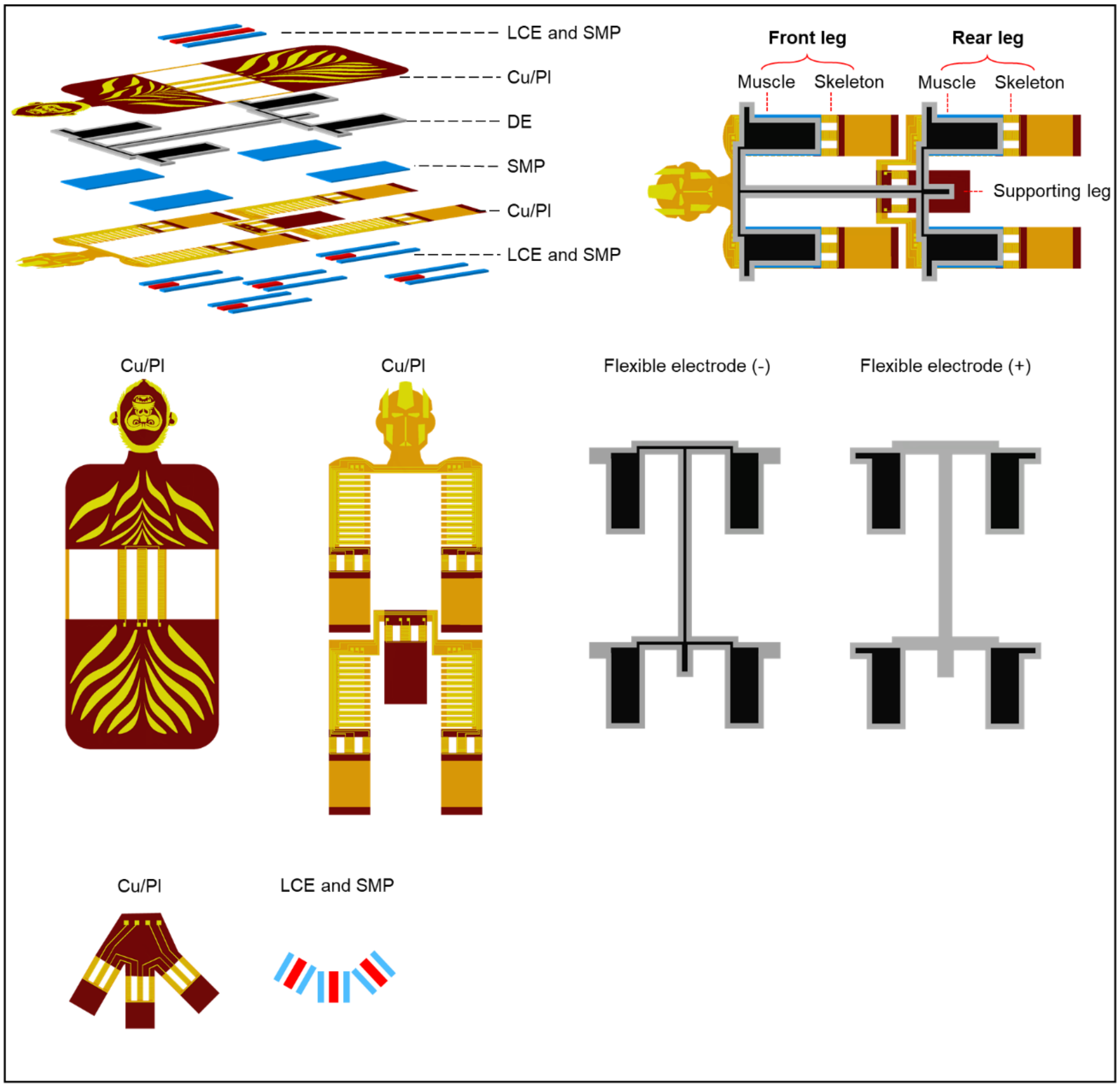

Fig. S19. Exploded view, Cu/PI pattern, LCE and SMP pattern, and DE pattern of the robot capable of transformation between quadruped and humanoid states.

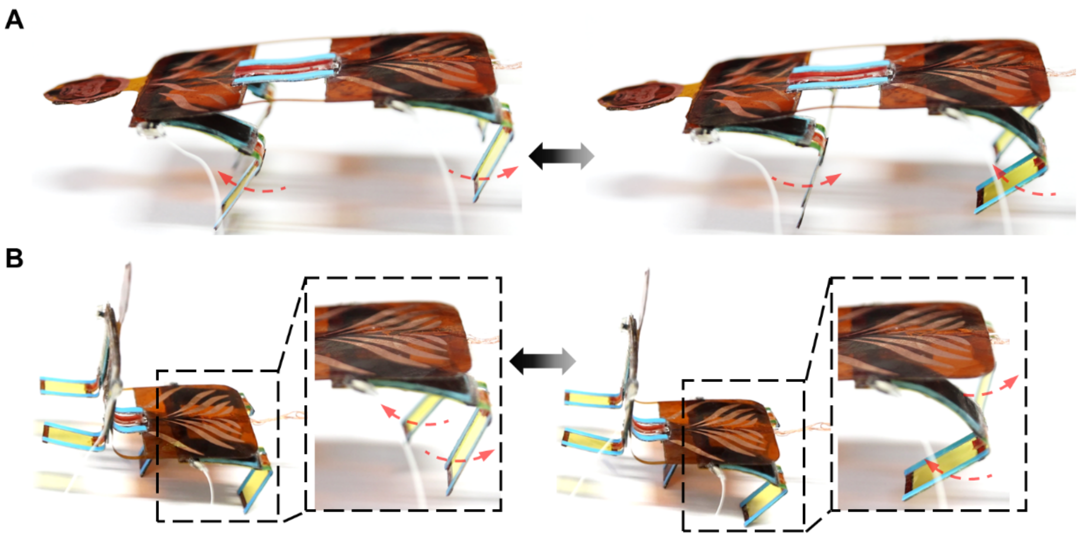

Fig. S20. Optical images showing locomotion modes of the morphable robot. (**A**) Quadruped locomotion driven by alternating actuation of the front and the rear muscles. **(B)** Humanoid locomotion driven by alternating actuation of the two rear muscle modules.

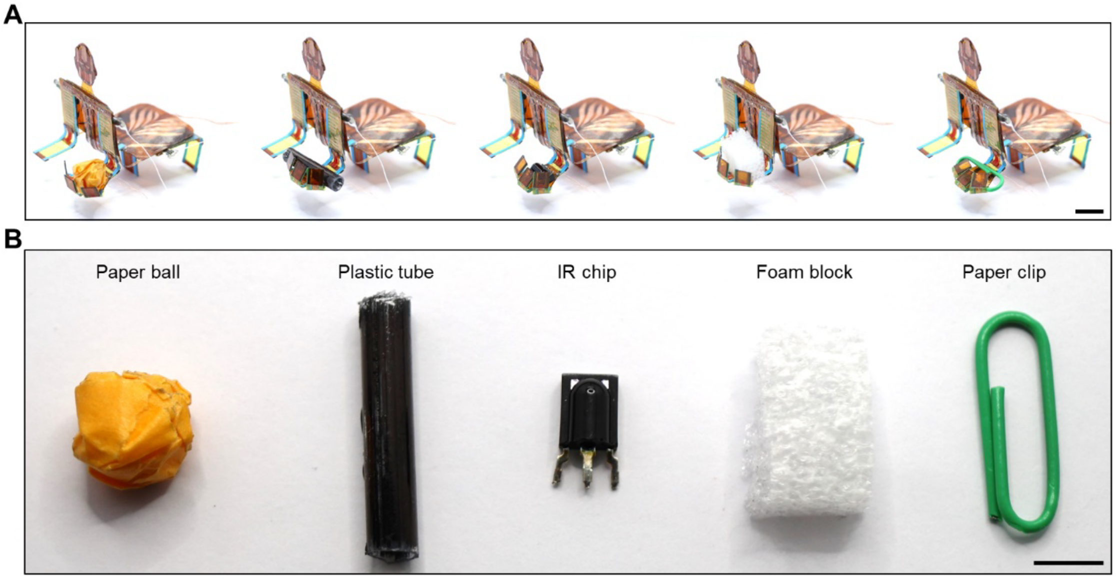

Fig. S21. Demonstration of functional extension via an additional gripper skeleton. (**A**) Optical images of the robot grasping different objects. Scale bar, 1 cm. (**B**) Objects of different shapes grasped by the gripper. Scale bar, 5 mm.

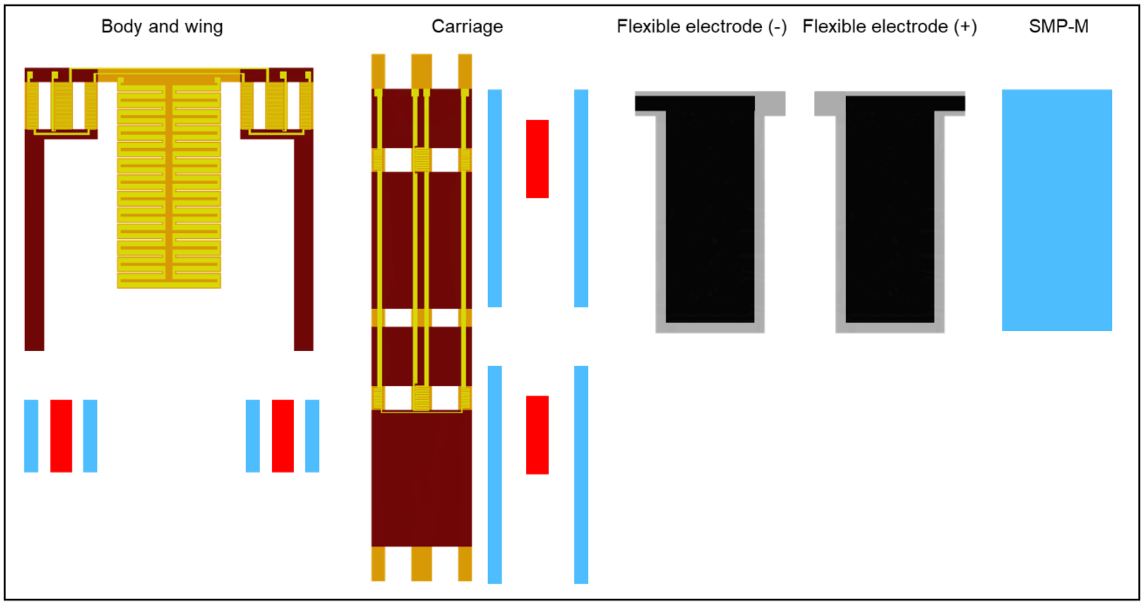

Fig. S22. Cu/PI pattern, LCE and SMP pattern, and DE pattern of the untethered robot.

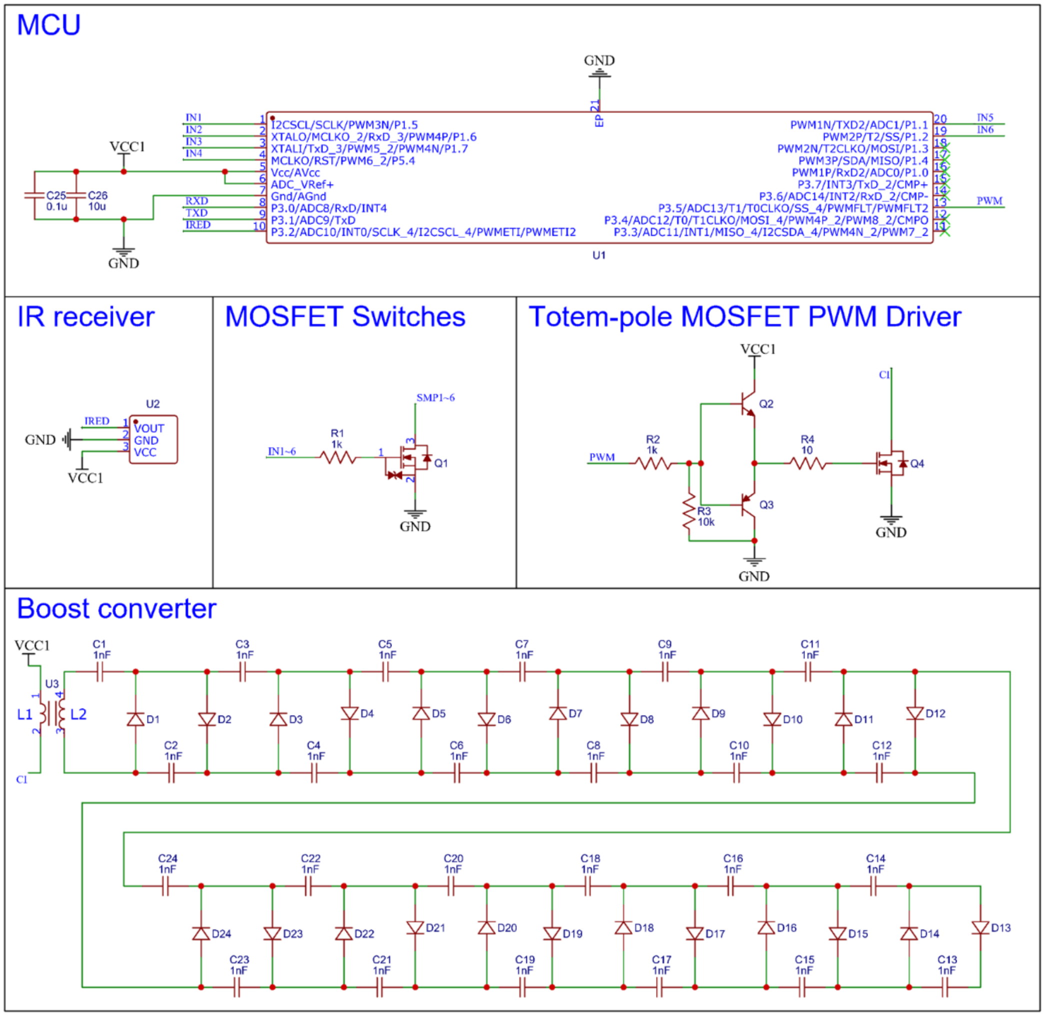

Fig. S23. Schematic diagram of ultralight control circuit. For clarity, only the import components of key modules are presented.

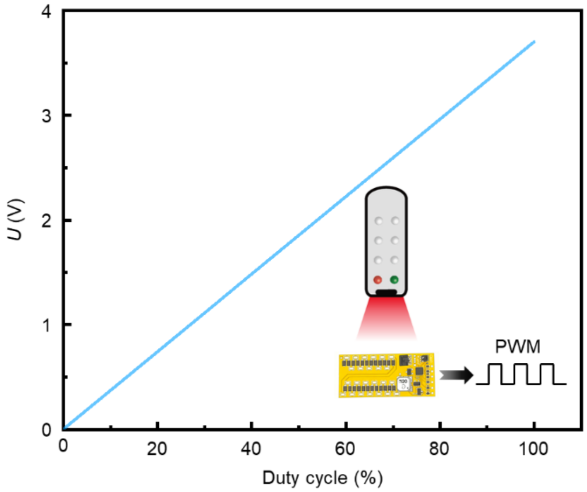

Fig. S24. The relationship between the output voltage applied to heating electrodes and the PWM duty cycle.

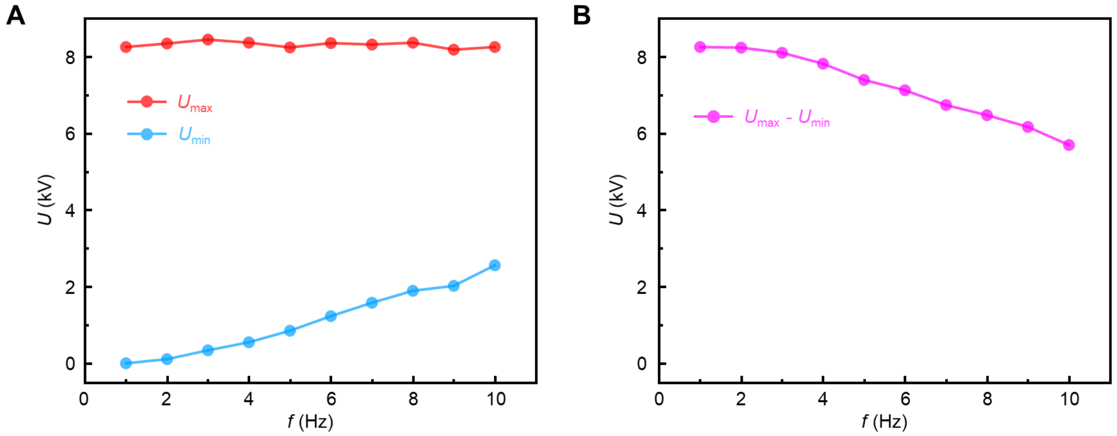

Fig. S25. HV output characteristics of the control circuit for the DE layer. (**A**) Maximum and minimum output voltages at different actuation frequencies. (**B**) Difference between the maximum and the minimum voltages as a function of actuation frequency.

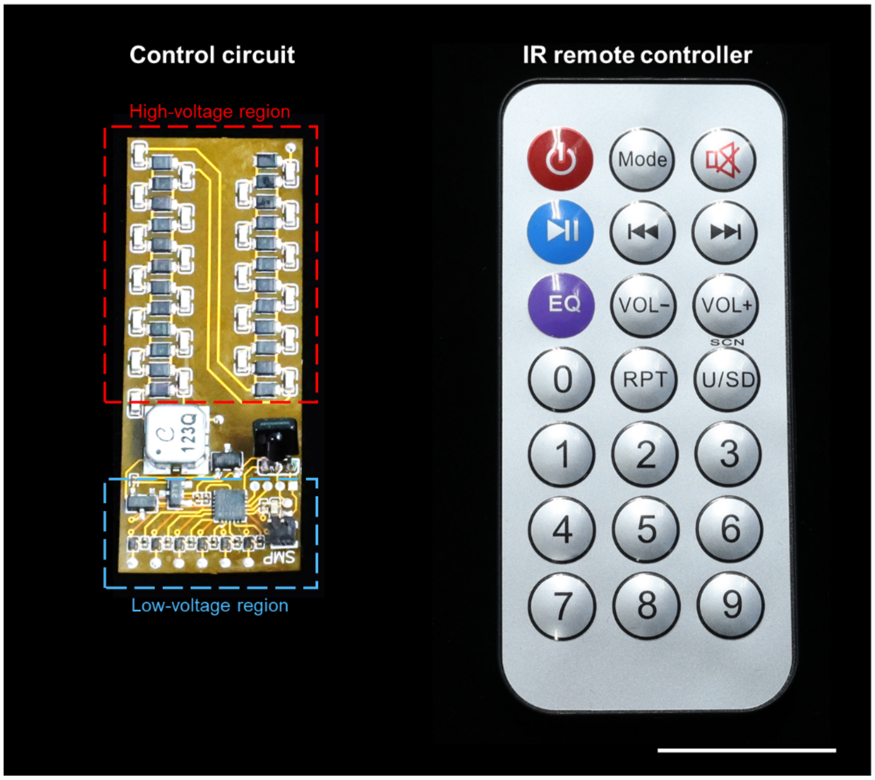

Fig. S26. Optical images of the control circuit and IR remote controller. Scale bar, 2 cm.

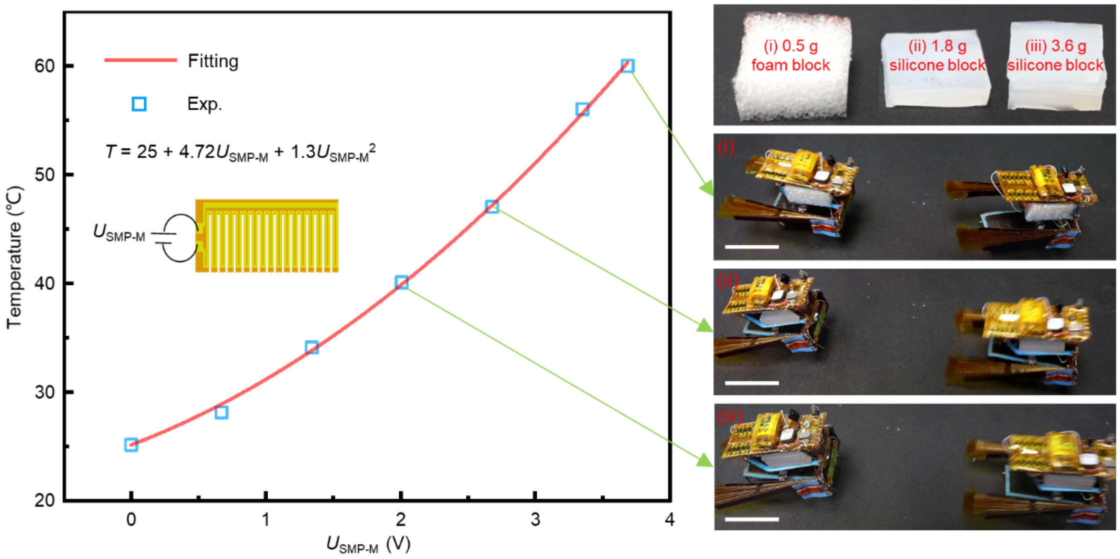

Fig. S27. Demonstration of cargo carrying under different loads enabled by stiffness modulation of the muscle module. The left panel shows the dependence of the temperature on the voltage applied to the SMP layer of the muscle module. Scale bars, 2 cm.

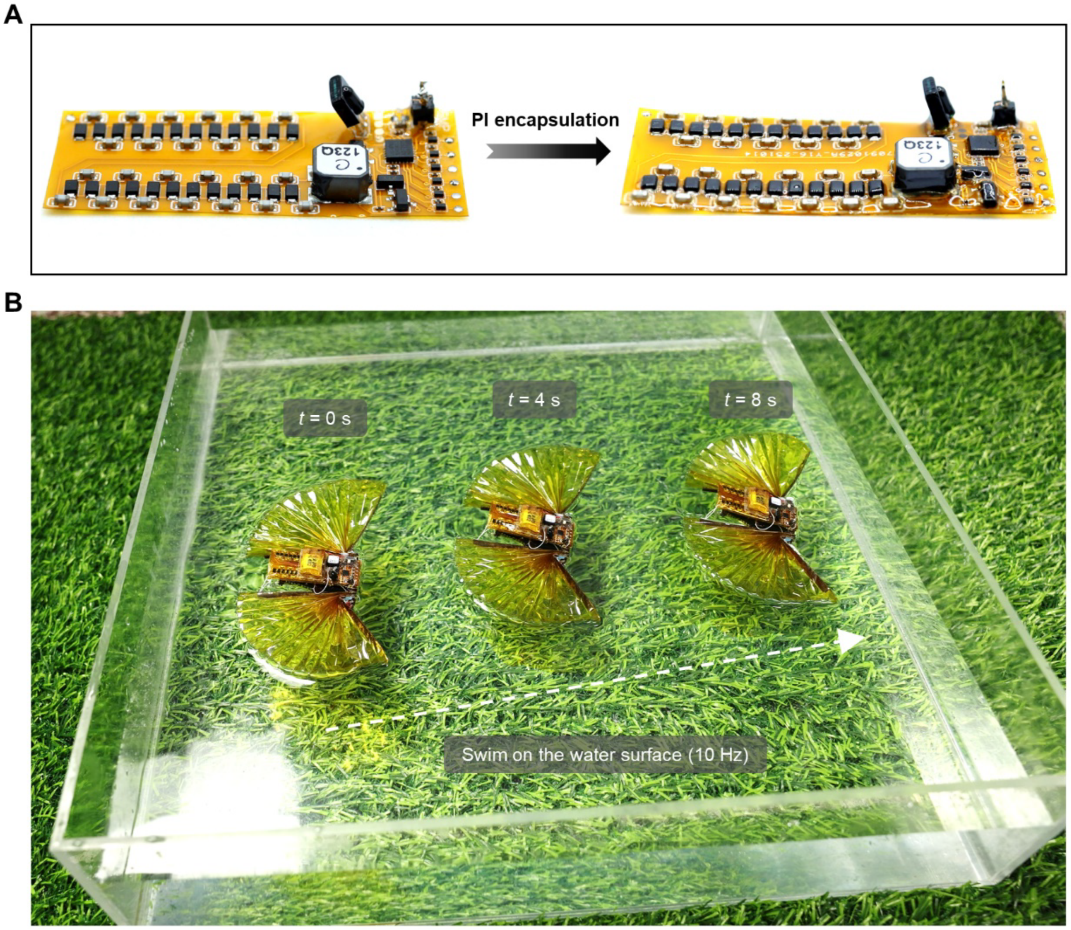

Fig. S28. Demonstration of water-surface swimming. (**A**) Encapsulation of the onboard circuit using a PI coating layer. (**B**) Swimming on the water surface at 10 Hz.

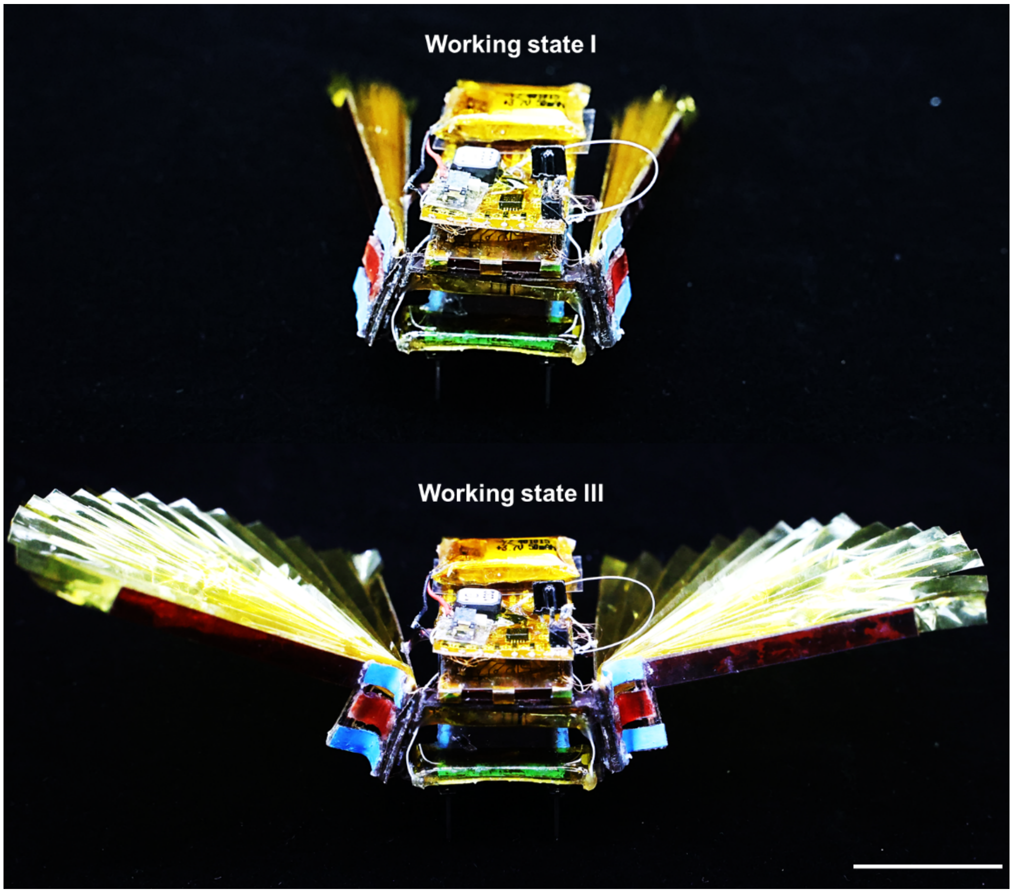

Fig. S29. Optical images showing the front view of the untethered robot. The wings on both sides are inclined upwards to generate sufficient lift during the gliding process. Scale bar, 2 cm.

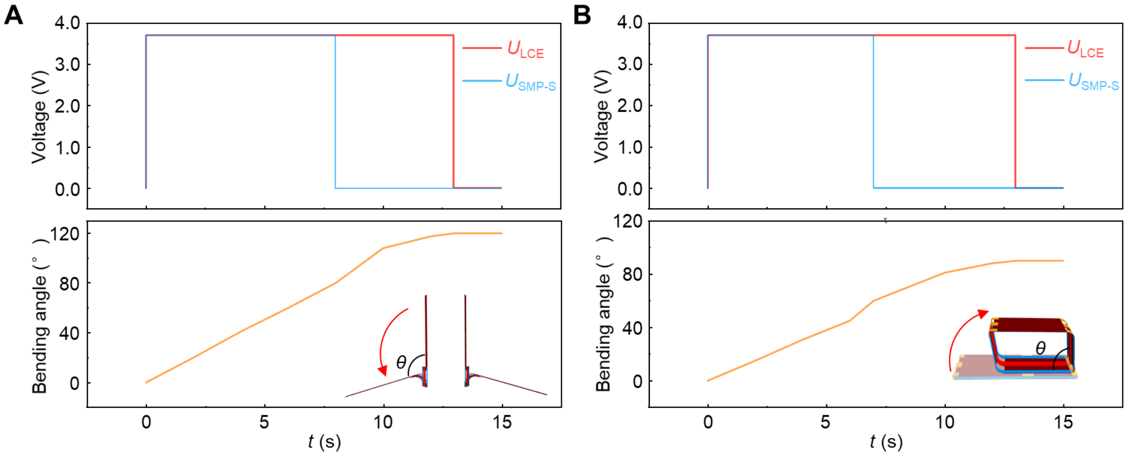

Fig. S30. Time histories of applied voltages and bending angles during the shape morphing and locking of the wings (A) and the carriage (B).

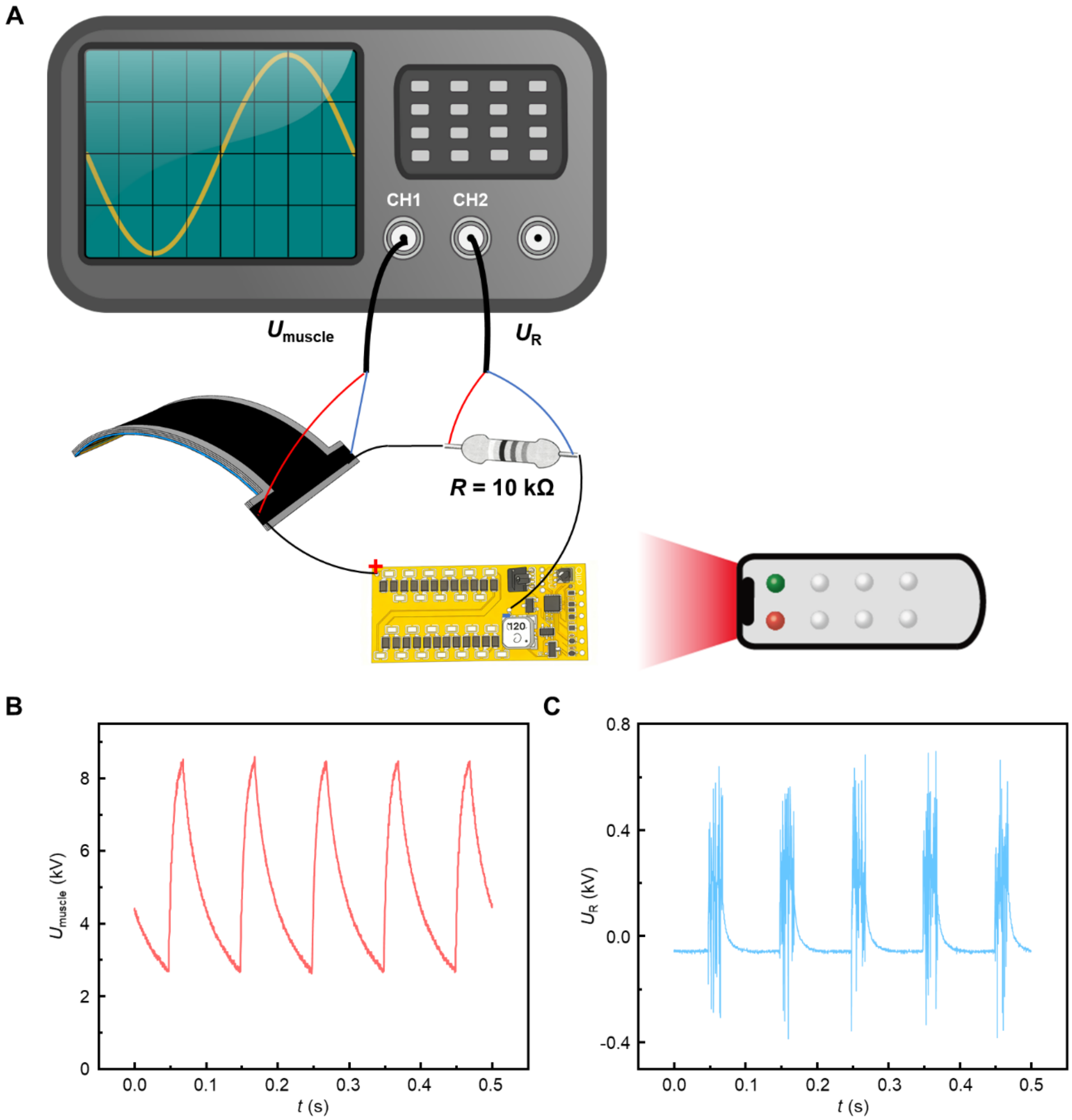

Fig. S31. Demonstration of the energy consumption measurements for the muscle module. (**A**) Schematic illustration of the experimental setup. (**B**) High voltage measured across the muscle module at 10 Hz in rigid state ($U_{SMP\text{-}M}$ = 0 V). (**C**) Voltage measured across the series resistor at 10 Hz in rigid state ($U_{SMP\text{-}M}$ = 0 V).

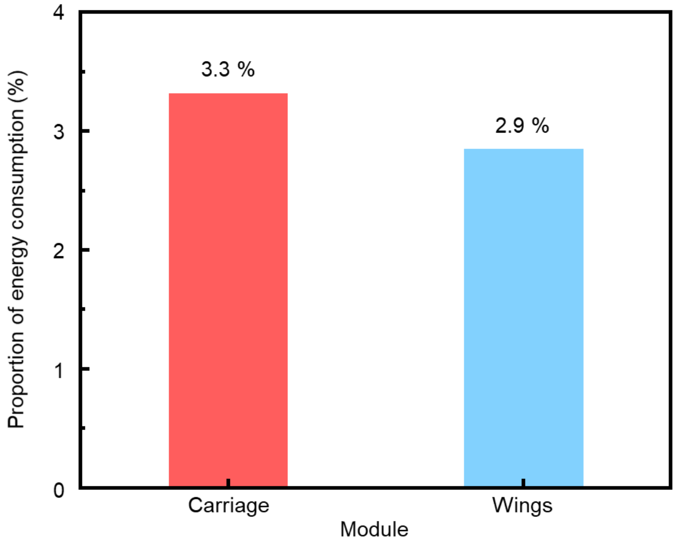

Fig. S32. Proportions of energy consumption for the carriage and wing transformations, in which a 3.7 V lithium battery with 50 mAh capacity is used.

Ref.	Size (cm)	Mass (g)	Untethered control	Morphing mechanism	Locomotion mechanism	Mode
This work	5	6.4	Yes	LCE/SMP-based electrothermal skeleton modules	Variable-stiffness DE-based muscle modules	Walk, glide, swim, and cargo-carrying
(11)	9	25	Yes	LCE/SMP-based electrothermal actuators	Motors	Terrestrial and aerial
(9)	25	900	Yes	Servo motors	Motors	Terrestrial and aerial
(8)	70	5600	Yes	Servo motors	Motors	Terrestrial and aerial
(12)	25	/	Yes	Electrothermal fiber-based actuators	Motors	Terrestrial and aquatic
(18)	25	2700	Yes	SMA-based electrothermal actuators	SMA-based electrothermal actuators	Swim, glide and crawl in deep-sea
(21)	15	/	No	Pneumatic actuators	Pneumatic actuators	Crawl and roll
(22)	15	/	No	SMA-based electrothermal actuators	SMA-based electrothermal actuators	Terrestrial and aquatic
(10)	40	9000	No	Pneumatic and electrothermal hybrid actuators	Motors	Terrestrial and aquatic

Table S1. Comparison of representative morphable robots.

Samples	E44 (mol)	D230 (mol)	T_g (℃)
SMP1	1.11	1	55
SMP2	1.22	1	63

Table S2. Compositions and properties of SMPs.

RM257 (g)	Toluene (g)	HHMP (g)	EDDET (g)	PETMP (g)	DPA (g)	T_{NI} (°C)
5	2.29	0.03	1.2	0.53	0.05	65

Table S3. Compositions and properties of LCEs.

Movie S1.
Design concept and principle of the musculoskeletal actuator. The muscle module integrates a multilayer PDMS-based DE with an electrothermally actuated SMP capable of stiffness change, enabling active modulation of resonant frequency, vibration amplitude, and actuation force. Serial integration with a morphable skeleton module capable of shape morphing and locking enables dual programmability in morphology and dynamics for the musculoskeletal actuator.

Movie S2.
Actuation performances of the muscle module. Bending responses of the muscle module under different actuation frequencies, high voltage applied to the DE, and stiffness states are demonstrated. The load-bearing capacities of the muscle module in both actuated and latched states are also shown.

Movie S3.
Actuation performances of the basic musculoskeletal actuator. Actuation performances under different muscle driving frequencies, muscle stiffness states, and skeleton configurations are demonstrated.

Movie S4.
Human-shaped musculoskeletal actuator. The centimeter-scale human-shaped musculoskeletal actuator is constructed from serially connected muscle and skeleton modules, demonstrating human-like actions (including kick, walk, run, jump, and swim) and load-bearing capability.

Movie S5.
Biomimetic musculoskeletal actuators with complex dynamic behaviors. Three representative actuators, including the peacock-like actuator, woodpecker-like actuator and scorpion-like actuator, are demonstrated, enabling the reproduction of complex dynamic behaviors of natural species.

Movie S6.
Morphable robot capable of transformation between quadruped and humanoid states. The shape transformation process, quadruped locomotion, and humanoid locomotion of the robot are demonstrated. The functional extension is further realized by attaching an additional gripper skeleton to the left front limb for object manipulation.

Movie S7.
Design and control of the untethered multimodal robot. The overall design concept and shape transformations of the robot are demonstrated. Maximum HV outputs of the onboard control circuit at different frequencies controlled by an IR remote controller are also shown.

Movie S8.
Terrestrial locomotion of the untethered multimodal robot at the undeployed state (state I). Slope climbing and locomotion under different actuation frequencies are demonstrated.

Movie S9.
Cargo-carrying capability of the untethered multimodal robot at the carriage-deployed state (state II). The robot is capable of carrying cargos of diverse shapes through controlled carriage deformation. Here, the increased muscle stiffness enables an enhanced load-bearing capacity.

Movie S10.
Locomotion of the untethered multimodal robot at the wing-expanded state (state III). Owing to the lightweight body and large deployable wing area, the robot can swim on the liquid surface (e.g., silicone oil and water). Besides, the inclined wings also generate sufficient lift to enable aerial gliding.
